# Observing Rivers With Varying Spatial Scales

**DOI:** 10.1029/2019WR026476

**Published:** 2020-09-14

**Authors:** Ernesto Rodríguez, Michael Durand, Renato Prata de Moraes Frasson

**Affiliations:** ^1^ Jet Propulsion Laboratory California Institute of Technology Pasadena CA USA; ^2^ Byrd Polar and Climate Research Center Ohio State University Columbus OH USA

## Abstract

The National Aeronautics and Space Administration/Centre national d’études spatiales Surface Water and Ocean Topography (SWOT) mission will estimate global river discharge using remote sensing. Synoptic remote sensing data extend in situ point measurements but, at any given point, are generally less accurate. We address two questions: *(1)What are the scales at which river dynamics can be observed, given spatial sampling and measurement noise characteristics? (2) Is there an equation whose variables are the averaged hydraulic quantities obtained by remote sensing and which describes the dynamics of spatially averaged rivers?* We use calibrated hydraulic models to examine the power spectra of the different terms in the momentum equation and conclude that the measurement of river slope sets the scale at which rivers can be observed. We introduce the *reach‐averaged Saint Venant equations* that involve only observable hydraulic variations and which parametrize within‐reach variability with a variability index that multiplies the friction coefficient and leads to an increased “effective” friction coefficient. An exact expression is derived for the increase in the effective friction coefficient, and we propose an approximation that requires only estimates of the hydraulic parameter variances. We validate the results using a large set of hydraulic models and find that the approximated variability index is most faithful when the river parameters obey lognormal statistics. The effective friction coefficient, which can vary from a few percent to more than 50% of the point friction coefficient, is proportional to the riverbed elevation variance and inversely proportional to the depth. This has significant implications for estimating discharge from SWOT  data.

## Key Points

- Remote sensing measurements of rivers, such as those from the SWOT mission, estimate quantities averaged over a reach, not at a point
- The Saint Venant equation is reformulated in terms of reach‐averaged variables, enabling estimation of river discharge from remote sensing
- The reach‐averaged equation contains an effective friction coefficient that depends on depth and width, which affects discharge estimation

## 1 Introduction

Remote sensing provides a novel approach for the estimation of hydraulic river parameters (Marcus & Fonstad, 2010), and global river data sets that use remotely sensed data are currently being developed by a growing community of hydrologists. Optical sensors, such as Landsat or MODIS, or microwave sensors, such as radars or radiometers, have been used to estimate global river width (Allen & Pavelsky, 2018; Smith, 1997; Smith & Pavelsky, 2008) and inundation extent (Brakenridge et al., 2012, 2007; Schumann et al., 2009). Digital elevation models (DEMs) derived from remote sensing data, such as HydroSHEDS (Lehner et al., 2008) or ArcticDEM (Morin et al., 2016), provide a snapshot of river surface elevation (e.g., Dai et al., 2018) and have been used to estimate slope and discharge (e.g., LeFavour & Alsdorf, 2005; Tuozzolo et al., 2019). For large rivers, dynamic estimates of water surface elevation are obtained using radar altimetry (see Cretaux et al., 2017; Ricko et al., 2012, for recent reviews) or, more recently, from lidar measurements (O'Loughlin et al., 2016; Zwally et al., 2012). Although these water surface elevation measurements are limited to cross sections located at the intersection of a river with the satellite nadir track, data from multiple altimeter or lidar missions are being compiled (e.g., the Hydroweb project, http://hydroweb.theia‐land.fr, or the ICESat derived inland water surface spot heights (IWSH) O'Loughlin et al., 2016; Zwally et al., 2012) to form a global data set of relatively sparse, but rapidly improving, water surface elevation measurements. Recently, hybrid data sets, combining remote sensing measurements from multiple sources, are being developed and used to improve estimates that use only a single sensor. Yamazaki et al. (2014) have developed the Global Width Database for Large Rivers (GWD‐LR), a data set that combines HydroSHEDS and SRTM imagery to provide global river widths collocated with DEM‐derived information. Sichangi et al. (2016) have combined river widths obtained from MODIS and altimetry data to improve on altimeter‐only discharge estimates.

In spite of the growing number of data sets, existing remote sensing measurements of hydraulic parameters are not yet sufficient for determining river discharge without additional in situ data, since this requires simultaneous measurements of multiple hydraulic variables. The upcoming NASA/CNES Surface Water and Ocean Topography (SWOT) mission (Alsdorf et al., 2007; Durand et al., 2010; Rodriguez et al., 2018), expected to launch in late 2021, will be the first mission to provide simultaneous measurements of river stage, width, and slope, allowing the possibility of river discharge estimates that do not require tuning with ground measurements (Durand et al., 2016).

A common theme in the estimation of river discharge from remote sensing measurement is the need to consider the spatial scale of the measurements and that is the subject of this paper. In theory, the equations governing river flow, such as the Saint Venant equations (Chow, 1959; Dingman, 2009), apply at a point, although it has been long recognized (e.g., Beven, 1989) that hydrologic parameters represent grid‐scale effective parameters, rather than true point measurements. In practice, (Barnes, 1967; Hicks & Mason, 1991), the parameter values have been calibrated using, when possible, homogeneous reaches that have nearly constant hydraulic parameters. Typical reach lengths are on the order of tens to hundreds of meters, although sometimes longer reaches are  used.

Remote sensing measurements have a finite spatial resolution and thus represent the average value over a distributed area (or length). The averaging scales are set by the capabilities of the sensor and often cannot be selected to guarantee that the hydraulic parameters will be constant over these scales. Since the hydraulic equations are not linear, and parameters may show significant within‐reach variability, it is not the case that spatial averages can be substituted for point measurements, as we will see below, and has been demonstrated using field data (Tuozzolo et al., 2019). Even if the measurement is collected with a spatial resolution sufficient to mimic a point measurement, the level of noise in the remote sensing measurement may be too large to ingest as a point measurement, and spatial averaging needs to be applied so the remote sensing measurements can be utilized meaningfully. This situation applies to the SWOT measurements: The elevation data will be densely sampled, but the elevation noise for a single measurement usually exceeds 50 cm and is not useful for the computation of water surface slopes. Another situation where spatial considerations must be taken into account is when the measurement has sufficient precision, but the sampling is sparse and the underlying dynamics cannot be represented fully. This is often the case with lidar or radar altimeter measurements.

In this paper, we address two questions central to the observation of rivers with measurements of varying spatial resolution and noise: *(1) What are the scales at which river dynamics can be observed, given spatial sampling and measurement noise characteristics? (2) Is there an equation, similar to the Saint Venant equation, whose variables are the averaged hydraulic quantities obtained by remote sensing and which describes the dynamics of spatially averaged rivers?*

To address the first question, we use the concept of *hydraulic visibility,* introduced by Garambois et al. (2017), defined as *the potential to depict a hydrological response and hydraulic variabilities within a river section or network via remote sensing*. As guidance in determining hydraulic visibility, we examine the ability of remote sensing observations to estimate the different terms in the Saint Venant equation at an appropriate signal‐to‐noise ratio. For oceanographic applications (Chelton et al., 2018; Rodriguez et al., 2018; Xu & Fu, 2012), a comparison of the power spectral density (PSD) (McCoy et al., 1998; Thomson, 1982) of the signal to that of the noise has been used successfully to define the ability to resolve spatial scales. Rivers are not stationary systems (i.e., river discharge and width vary systematically when moving in the downstream direction), but we assume that changes in river characteristics are slow enough so that a PSD makes sense over limited stretches of a river. We can then observe the distribution of spatial scales characteristic of the flow for each river in our study and compare them against measurement noise characteristics from potential remote sensing sensors. Although the use of Fourier spectra to examine river dynamics is not common, they have been used fruitfully by Horritt (2002) and Li et al. (1992) to examine the scales of response of water surface elevation to riverbed variations.

Once it has been established that terms in the dynamic equation can only be observed at scales greater than the limit imposed by the measurement noise, a process must be established to ingest the remote sensing data at a suitable scale. One approach for doing this is to filter the measurements spatially over a homogeneous reach, a process we call *reach averaging*, until a scale is reached (the *hydraulic visibility scale)* such that the PSD of the filtered signal lies above the PSD of the noise. The filtered variables will then be *hydraulically visible* but cannot be used directly in the dynamic equation, due to the nonlinearity of the friction and dissipation terms. As an example of the problem, consider a riffle and pool sequence contained in a river reach, such that the scale of the pools and riffles is much smaller than the hydraulic visibility scale. The reach‐averaged parameters would properly exhibit the change in potential energy, but the dissipation of kinetic energy through friction with the variable bottom would not be observable at all, and the dynamic equations for the *“average river”* would be representative of faster river flow with a larger Froude number (Dingman, 2009). Below, we show that it is possible to obtain a dynamic equation for the average flow, provided we parametrize suitably the energy loss parameters, the friction coefficient, and the dissipation of kinetic energy head, to account for the variation of hydraulic parameters within the reach. This sort of rescaling is familiar from the Boussinesq term (Boussinesq, 1877; Chow, 1959) in the Saint Venant momentum equation, which multiplies the kinetic energy dissipation term and which parametrizes the *vertical* variability of the river current in the one‐dimensional flow approximation. In our approach, a similar term arises to account for the *horizontal* variability of the kinetic energy.

The friction coefficient, such as Manning's *n*, is not a direct observable and is usually calibrated experimentally for each reach, given the other hydraulic variables and a flow law, such as the Manning equation, to match the observed discharge. In this paper, we develop a theoretical approach to understand the influence of channel variability on flow resistance in a natural river reach. For the reach‐averaged flow, we find that these friction coefficients cannot be used with the flow law containing the average parameters. The losses due to friction must be parametrized with an increased friction coefficient to reflect nonlinear interactions between the the hydraulic parameters that determine the friction slope.

In a previous study, Li et al. (1992) used a perturbation approach, similar to the one presented in Appendix A1, to study the impact of channel variability on the effective friction coefficient, compared to that in a smooth channel. The present study expands and complements the work of Li et al. (1992) by deriving an *exact* expression for the effective friction coefficient, given subreach variabilities. It also provides a detailed comparison of the predicted effect against calibrated models and predicts its dependence on the river depth.

While the discharge relations at a station show significant variability, it has been argued (e.g., Jowett, 1998; Navratil & Albert, 2010) that hydraulic relations between river parameters (Leopold & Maddock, 1953) are more stable at a reach level. Smith and Pavelsky (2008) have argued that the incorporation of remotely sensed hydrologic data needs to move away from the point‐based approach used for in situ measurements of discharge to an approach that uses reach‐averaged measurements. Using optical remote sensing data of the Lena River, they demonstrated the ability to predict discharge using reach‐averaged river width. In fact, the coefficient of the power law relation between width and discharge stabilized once sufficient reach averaging of the river width was performed. This hints at the possibility that hydraulic geometry relations might be best viewed as statistical relationships applying to the ensemble present in a river reach, rather than at a cross section.

One of the benefits of the reach‐averaged dynamic equation we derive is the ability to obtain consistent dynamics when upscaling high‐resolution observations to lower‐resolution models. This process is typical of routing models, such as the Catchment‐Based Macro‐scale Floodplain (CaMa‐Flood) model (Yamazaki et al., 2011), that are used as components for large‐scale hydrologic models, such as the Variable Infiltration Capacity (VIC) land surface hydrological model (Liang et al., 1994). The assimilation of remotely sensed data into these models also requires going consistently from high‐ to low‐resolution models, and the equations derived here provide a consistent methodology for upscaling models and observations.

We use a large representative data set of rivers to demonstrate and validate the concepts discussed above. The river dynamics are based on calibrated hydraulic models that utilize real discharge measurements and hydraulic parameters: We discuss the characteristics of the data in section 2. In section 3, the hydraulic visibility of the Saint Venant equation is examined by computing the PSD of its components and comparing against different sensor noise levels. We conclude that the visibility of the friction slope represents the greatest challenge, since the computation of the slope from noisy elevations amplifies the noise at small distances. To overcome this limitation, we introduce the reach‐averaged Saint Venant equation and show that it is formally nearly identical to the original equation but requires the inclusion of terms that characterize the variability of the hydraulic parameters within the reach. The parametrization of the friction slope is the most significant change that must be included, and in section 4.3, we show that within‐reach variability of the hydraulic parameters can be included by rescaling the friction coefficient by a factor proportional to the total within‐reach variance of the parameters. To illustrate the concepts, we introduce in section 5.2 a simple model for a riffle and pool sequence that can be solved exactly and at arbitrary spatial resolution for gradually varied flow. A study of more complicated river conditions is then presented in section 5.3, where we characterize the changes in the friction coefficient that are observed by reach averaging the calibrated hydraulic models and compare the exact results with simple estimates that are obtained assuming a lognormal distribution of hydraulic parameters, as been suggested by Moody and Troutman (2002) and more recently by Allen et al. (2018). We show that this simplifying assumption provides reasonable estimates for changes in the friction coefficient which do not require detailed knowledge at scales smaller than a river reach. It is useful to correct for this variability using only observable parameters Using a simple analytic model, we derive the dependence of the index of variability on river depth and bathymetry fluctuations. We conclude by summarizing our results and drawing some conclusions about the upcoming SWOT mission river discharge.

## 2 Data Sets Used

In order to evaluate our results, we have used the set of 24 hydraulic model applications listed in Table 1. A subset of these models was used in the evaluation of discharge algorithms for the NASA SWOT mission (Durand et al., 2016), and additional model details can be found there. The hydraulic variables used in the models were collected from field measurements, and the friction coefficient was calibrated to match discharge measurements over periods extending from a few months to a year that included dynamics varying from low to high discharge conditions. The model physics was assumed to be described by one‐dimensional flow and included dynamic terms in the solution of the momentum equations for all models. Most of the models used the Army Corps of Engineers HEC‐RAS model (Brunner, 2016), but LISFLOOD‐FP (Bates et al., 2010, 2013), MASCARET (Goutal et al., 2012), and ProSe (Even et al., 1998) were also used, as shown in Table 1. We note that some of the models (e.g., LISFLOOD‐FP) are intrinsically two‐dimensional, but the model output for this study has been converted to one‐dimensional average parameters to allow comparisons with other models. Another limitation of the cases utilized is the fact that, in general, the Manning friction coefficient did not vary significantly but was calibrated to match the flow globally. Future tests of this work will use field  data.

**Table 1:** Some Statistics for the Rivers and Reaches Used in This Study, Complimentary to Figure 1

	$\bar{Q}$		*L*		$\bar{L_{r}}$		
River name	m^3^/s	*F*_*r*_	km	*N*_*r*_	km	Model	Reference
Severn	63.7	0.11	68.2	4	6.3	LISFLOOD‐FP	Neal et al. (2015)
Seine upstream	126.8	0.03	84.6	4	9.6	ProSe	Even et al. (1998)
Garonne upstream	128.5	0.23	75.2	16	4.1	HEC‐RAS	Larnier (2010)
Sacramento upstream	181.1	0.16	74.9	7	9.6	HEC‐RAS	Rogers (2014)
Sacramento downstream	212.7	0.09	36.2	9	3.6	HEC‐RAS	Rogers (2014)
Seine downstream	223.7	0.04	128.1	4	29.0	ProSe	Even et al. (1998)
Arial Khan	407.0	0.06	104.9	10	9.8	HEC‐RAS	Siddique‐E‐Akbor et al. (2011)
Garonne downstream	479.9	0.19	49.0	8	5.5	MASCARET	Larnier (2010)
Kanawha	579.3	0.05	91.0	4	3.8	HEC‐RAS	Adams et al. (2010)
Kushiyara	737.8	0.08	264.9	5	50.2	HEC‐RAS	Maswood and Hossain (2016)
Cumberland	935.5	0.08	42.6	4	8.6	HEC‐RAS	Adams et al. (2010)
Ohio section 1	1,336.1	0.05	96.2	9	9.7	HEC‐RAS	Adams et al. (2010)
Ohio section 7	4,705.5	0.07	71.8	7	8.4	HEC‐RAS	Adams et al. (2010)
Ohio section 2	2,073.8	0.07	88.1	8	9.7	HEC‐RAS	Adams et al. (2010)
Ohio section 3	2,496.1	0.08	140.8	14	9.7	HEC‐RAS	Adams et al. (2010)
Ohio section 4	2,851.4	0.05	51.5	5	9.3	HEC‐RAS	Adams et al. (2010)
Ohio section 5	3,273.0	0.08	86.5	8	9.2	HEC‐RAS	Adams et al. (2010)
Ganges	4,540.3	0.14	230.2	6	15.8	HEC‐RAS	Siddique‐E‐Akbor et al. (2011)
							Maswood and Hossain (2016)
Mississippi upstream	4,893.8	0.15	175.4	3	15.8	HEC‐RAS	Adams et al. (2010)
Mississippi intermediate	4,906.8	0.16	95.1	9	9.3	HEC‐RAS	Adams et al. (2010)
Ohio section 8	6,307.1	0.10	71.2	6	8.0	HEC‐RAS	Adams et al. (2010)
Mississippi downstream	14,202.5	0.14	173.0	6	29.4	HEC‐RAS	Adams et al. (2010)
Padma	22,111.9	0.12	105.1	5	14.3	HEC‐RAS	Siddique‐E‐Akbor et al. (2011)

*Note*. $\bar{Q}$ is the median discharge, *F*_*r*_ is the median Froude number, *L* is the median centerline length, *N*_*r*_ is the number of reaches, and $\bar{L_{r}}$ is the median reach length.

The rivers selected for this study were chosen to span a large variety of flow conditions and river morphologies, although we concentrated on rivers large enough to be amenable to observation with spaceborne remote sensing instruments, which currently sets a limit on the river width to be on the order of 50–100 m. The rivers were in order of median discharge: the Severn, the longest river in Great Britain; the Seine (upstream and downstream sections), which drains the north of France; upstream and downstream sections of the Garonne River, which drains a large section of south west France; upstream and downstream reaches of the Sacramento River, in California; the Arial Khan River, one of the main southeastward outlets of the river Padma, in Bangladesh; the Kanawha River, a tributary of the Ohio River, in West Virginia; the Kushiyara River, a distributary river in Bangladesh and Assam, India; the Cumberland River, a major tributary of the Ohio River; the Ohio River, the largest tributary to the Mississippi River; the Ganges, the major river draining Northern India and Bangladesh; the Mississippi River, the largest river in the United States; and finally, the Padma River, the combined flow of the Ganges and Jamuna rivers after their confluence at Goalandaghat. We select the homogeneous river segments listed Table 1, which range in length from ∼36 km, for the smaller rivers, to ∼265 km, for the largest ones. These segments are selected to span a wide range of hydraulic conditions. The median Froude number, *F*_*r*_, is smaller than 0.25, so that the flow is generally subcritical.

**Figure 1:**
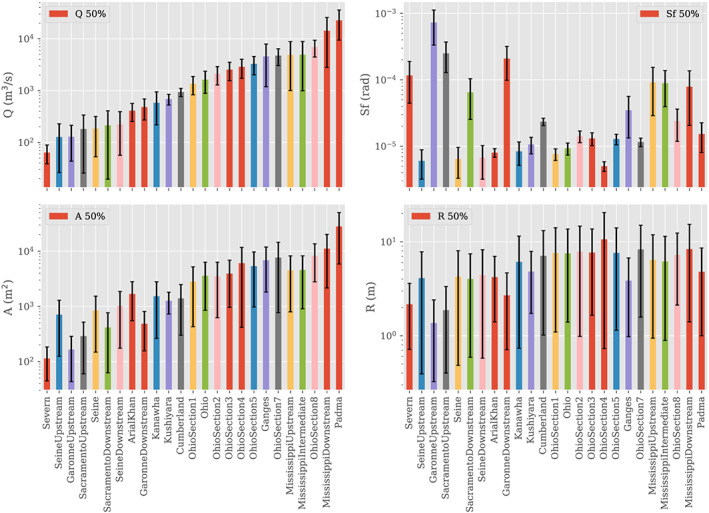
Hydraulic parameter ranges for the river models used in this paper: (upper left) *Q*, the river discharge; (upper right) *S*_*f*_, the friction coefficient; (lower left) *A*, the wetted cross section; and (lower right) *R*, the hydraulic radius. The median values are represented by color bars, and the minimum to maximum range is indicated by the black error bars.

In Figure 1, we present the median values, as well parameter ranges, for the basic hydraulic variables used in this study. Every model in this study reports discharge, *Q*, wetted cross section, *A*, Manning's friction coefficient, *n*, river width, *W*, water surface elevation, *h*, and thalweg depth, *H*, at stations separated by distances that range from less than 100 m, for the smaller rivers, to over 1 km, for the larger rivers. From the time history of *A*, *W*, *h*, and *H*, it is possible to estimate the river velocity, *U*=*Q*/*A*, the wetted perimeter, *P* (assuming a single channel), the hydraulic radius, *R*=*A*/*P*, and the riverbed bathymetry, *Z*_0_. The friction slope, *S*_*f*_, can then be estimated assuming that the Manning equation is an appropriate description of the bed stress (Dingman, 2009):

(1) $$S_{f}=\frac{n^{2}U^{2}}{R^{4/3}}=\frac{n^{2}Q^{2}}{A^{2}R^{4/3}}$$

In this study, we concentrate on the variables *Q*, *A*, *R*, *∂*_*x*_ *h*, and *S*_*f*_ since their behavior specifies the Saint Venant equation (Dingman, 2009), and they are amenable to remote sensing observations, as described below. The derivative of the surface water elevation in the downstream (*x*) direction is estimated from water surface elevation, *h*, reported at each station and may be underestimated if the model cross sections are at too great a separation. The discharge, *Q*, ranges 3 orders of magnitude, from ∼20 m^3^/s to ∼3 ×10^4^ m^3^/s, with strong correlation and a similar span of ranges for the wetted cross section, *A*. The hydraulic radius, *R*, has a weaker dependence on discharge and ranges from below ∼1 m to a bit above 10 m. Finally, the friction slope, *S*_*f*_, has the greatest fractional variability and no visible correlation with the discharge.

To perform reach averaging, the river segments are broken up into homogeneous reaches whose average lengths are given Table 1. The reach lengths vary from 3–5 km, for the more variable smaller rivers, to as large as 50 km, for the larger rivers. The criteria for reach selection are discussed in detail in Durand et al. (2016) and Frasson et al. (2017) and include the presence of flow control points, changes in flow profiles, and river sinuosity.

## 3 Hydraulic Visibility of the Saint Venant Equation

In section 1 we saw that remote sensing data are being used actively to estimate river discharge. However, most of these approaches use remote sensing data to complement in situ data, such as calibrated rating curves from altimetry (Cretaux et al., 2017), statistical hydraulic relations (Andreadis et al., 2013; Bjerklie, 2007; Bjerklie et al., 2003, 2018; Gleason et al., 2014), or by assimilating water level data into existing models (Andreadis et al., 2007; Biancamaria et al., 2010; Brisset et al., 2018; Durand et al., 2008; Oubanas et al., 2018). While these approaches provide useful information when suitable complimentary information is available, there are many areas of the world where this information is not available (Alsdorf et al., 2007; Pavelsky et al., 2014) and approaches that use remote sensing as the only input data for estimating river discharge become attractive. Motivated by the upcoming SWOT mission, new approaches have been proposed to estimate river discharge using only a (potentially long) time series of remote sensing data. In addition to approaches that make assumptions about hydraulic geometry, approaches have also emerged (Durand et al., 2010, 2014; Garambois & Monnier, 2015; Gleason et al., 2017; Yoon et al., 2016) that use an approximate form of the Saint Venant equation to invert for discharge, along with hydraulic variables that are not directly observed. The relative advantage of these approaches is an active area of investigation (Bonnema et al., 2016; Durand et al., 2016).

Here, we follow the approaches that use the Saint Venant equation and study explicitly the spatial dependence assumptions that were implicit in previous studies. We examine hydraulic visibility by starting from the Saint Venant continuity (equation  2) and momentum conservation (equation  3) equations, together with the Manning parametrization of the friction slope (equation  1) (although, as discussed in the next section, other parametrizations can also be included in our approach). The momentum equation is written such that the hydraulic visibility of each term is emphasized and is derived and discussed in greater detail in section S1 of the supporting information.

(2) $$q=\frac{\partial A}{\partial t}+\frac{\partial Q}{\partial x}$$

(3) $$\underset{\text{Friction}\;\text{Slope}}{\underbrace{S_{f}}}=\underset{\text{Surface}\;\text{Slope}}{\underbrace{-\frac{\partial h}{\partial x}}}-\underset{\text{KE}\;\text{Gradient}}{\underbrace{\frac{\beta }{2g}\frac{\partial U^{2}}{\partial x}}}-\underset{\text{Unsteady}+\;\text{Inflow}}{\underbrace{\frac{1}{gA}\left[\frac{\partial Q}{\partial t}+U\frac{\partial Q}{\partial x}\right]}}$$

In addition to the previously defined symbols, we introduce *q*, the lateral discharge, and *β*, the Boussinesq momentum term that parametrizes the *vertical* current variability contribution to the advected momentum (Boussinesq, 1877; Chow, 1959; Dingman, 2009). Following Ponce and Simons (1977), and subsequent authors (Hunter et al., 2007; Moramarco & Singh, 2002), we order the terms by the magnitude of their expected contribution to the friction slope for flows that are slowly varying. The first term, *∂*_*x*_ *h*=−*S*_0_+*∂*_*x*_ *H*, is the total water surface slope, which includes contributions from the bed slope, *S*_0_, and the pressure term due to changes in flow depth, *H*. Retaining only the first term leads to the *diffusion wave* approximation; including the next term leads to the *steady dynamic wave* approximation, while keeping all terms has the full *dynamic wave*. Notice that using SWOT‐like data, using the *kinematic wave* approximation, *S*_*f*_≈*S*_0_, is not possible, since the bed slope and flow depth are not observed by measuring the water level, but the diffusion wave approximation, which is more accurate, can be used to estimate the discharge.

The second term is the kinetic energy head gradient and accounts for losses in kinetic energy downstream. This term arises from the term proportional to *U∂*_*x*_ *U* in the Saint Venant equation and can also be thought of as the advective inertia term in the total derivative of the velocity. Hunter et al. (2007) examine the relative contribution of this term and conclude that *[a]dvection forces will come to dominate for small length scale features (which generate significant velocity derivatives), but at larger length scales, bed friction will dominate and the advection terms may be neglected*. They estimate that this term becomes important when the spatial scales are of order 100 m but is negligible for larger scales. Almeida and Bates (2013) also note the importance of this term as the Froude number increases, leading to steeper gradients than those predicted by the diffusion wave approximation for subcritical flow and predicting depth gradients of the opposite sign to the diffusion wave approximation and contributing to unsteady  flow.

Finally, the unsteady and lateral flows are contained in the last term. The ability to recover dynamic events depends on appropriate temporal sampling and will be missed at reach level by the poor temporal sampling typical of spaceborne missions. Temporally sparse remote sensing data can still be useful to basin‐wide assimilation (e.g., Biancamaria et al., 2010).

Here, we concentrate on hydraulic visibility at the reach level, rather than the basin level, where assimilation will be required. The ability to recover reach‐level discharge from pass data is of importance to many applications. The reach‐average discussion presented here is still relevant to the full unsteady problem, when adequate temporal sampling is available (e.g., during the SWOT fast sampling phase).

A closer connection with quantities amenable to remote sensing is obtained by combining the momentum and continuity equations, to obtain the following equation for the friction slope:

(4) $$S_{f}=-\frac{\partial h}{\partial x}-\beta \frac{\partial }{\partial x}\frac{U^{2}}{2g}-\frac{1}{g}\left[\frac{\partial U}{\partial t}+\frac{Uq}{A}\right]$$

A strategy for using these equations for a discharge algorithm to estimate the discharge purely from remote sensing observations is to estimate *S*_*f*_ using observations of surface slope and river velocity (and their derivatives) and to use the Manning equation, together with estimates of *R*, *A*, and *n* to estimate the discharge. At this time, no single instrument can make all of these measurements. River currents and width have been estimated remotely using along‐track interferometry (Bjerklie et al., 2005; Romeiser et al., 2007) or optical tracking of sediments (e.g., Pavelsky & Smith, 2009), but these techniques suffer from weather and sediment concentration limitations (optical imaging) or do not yet figure in the plans for future spaceborne missions (radar). Radar or lidar altimeters can measure surface water elevation but, due to the separation between tracks, cannot measure slope well, except for large rivers.

The SWOT mission (Alsdorf et al., 2007; Durand et al., 2010; Rodriguez et al., 2018) offers the best chance, at this time, to observe most of the terms in these equations. SWOT will provide high‐resolution imaging of rivers together with dense, although noisy, surface water elevation measurements. If the river is observed over its annual cycle, the channel bathymetry can be reconstructed by using measured water levels and the observed water extent from radar images to form a contour map of the channel above minimum flow. The observed wetted cross section above the lowest level, *δA*, can be obtained from the estimated bathymetry above the minimum level and the measured elevations. To obtain the total cross section, *A*=*A*_0_+*δA*, the observed river dynamics must be used to estimate the unobserved wetted cross section, *A*_0_. Several authors have demonstrated algorithms for estimating *A*_0_, together with a reach‐averaged Manning friction coefficient, *n*, using least squares (Garambois & Monnier, 2015) or Monte Carlo (Durand et al., 2014, 2016; Yoon et al., 2016) minimization, by fitting hydraulic geometry relations (Gleason et al., 2014), or inverting stage‐discharge relations (Bjerklie et al., 2018; Durand et al., 2016). Combining the estimated *A*_0_, which remains constant throughout the mission, with *δA* and width, *W*, measurements for each satellite pass, the total wetted cross section, *A*, and hydraulic radius, *R*≈*A*/*W*, can be obtained. The water surface slope, *∂h*/*∂x*, is estimated by performing a linear fit in the downstream direction to the measured elevations. Assuming the diffusion wave approximation (Ponce & Simons, 1977), as in Durand et al., 2014, 2010), Garambois and Monnier (2015), and Yoon et al. (2016), one estimates the friction slope by keeping only the first term on the right‐hand side of equation (4): *S*_*f*_≈−*∂h*/*∂x*. The discharge, *Q*, and velocity, *U*, can then be estimated using Manning's equation (1) together with the estimates of *n* and *R* obtained by estimation using the observed variability of hydraulic variables, as described above.

One can go beyond the diffusion wave approximation by using Manning's equation to estimate *U* and using this estimate to calculate *∂*_*x*_ *U* ^2^ in the momentum equation to get the steady dynamic wave approximation (Ponce & Simons, 1977) for *S*_*f*_. The process can be iterated until convergence is achieved to a suitable accuracy. However, while the process works for most noise‐free data, the presence of noise is magnified by the derivatives and may lead to a lack of convergence.

The previous discussion did not account for measurement noise. In the presence of noise, useful estimates can only be obtained after reducing the noise level by averaging independent estimates along a reach. Given a measurement noise level, how long should a reach be to suppress the noise and recover the signal? Montazem et al. (2019) propose a wavelet‐based decomposition to recover river control points while balancing measurement noise.

We suggest that, since the spectral characteristics of the measurement error are known (Rodriguez et al., 2018), a suitable criterion is to require that the averaging scale be such that the PSD of the filtered signal is greater than the noise spectral density. The PSD describes the contribution to the total signal variance as a function of spatial frequency and is defined as the expected value of the square modulus of the signal's Fourier transform. For stationary processes, it can be shown to be equal to the Fourier transform of the signal spatial covariance function. The estimation of the PSD from experimental data has a long history, since it is a noisy process. A good method for obtaining reduced variance from geophysical data is provided by the multitaper PSD estimator (Thomson, 1982). As discussed by McCoy et al. (1998), the multitaper spectral method is appropriate for estimating PSD for geophysical variables characterized by power law behavior due to their ability to reduce spectral variance at higher spatial frequencies.

The model data for this study (Frasson et al., 2019) contain time series of the hydraulic parameters for rivers that have been split into homogeneous reaches (Frasson et al., 2017). For each parameter and each reach, we linearly interpolate the hydraulic variables to a uniform spatial grid of spacing equal to the median model *x* spacing. The derivative terms, *∂*_*x*_ *h* and *∂*_*x*_ *U* ^2^, were calculated with single step, backward difference derivatives, to be consistent with the method for solving the steady gradually varying flow equation, described below. However, the results did not change significantly if centered, forward, or spline interpolation derivatives were used. Equation  1 was used to calculate the friction slope, *S*_*f*_, using the model hydraulic variables at each point. For each time, we remove a linear trend over the reach and use the Python Nitime library (http://nipy.org/nitime/) to compute the multitaper PSD estimator (Thomson, 1982) of the spatial variability of the hydraulic variable. In the figures below, we plot the median spectrum of the spectrum time series (solid lines). The stability of the spectra over time is shown by shading the region containing 50% of the data in the second and third quartiles of the PSD values at each spatial frequency. These spectra are plotted against 1/*λ*, where *λ*=2*π*/*k* is the wavelength characteristic of the PSD spatial angular frequency, *k*.

For altimeters and interferometers, the surface water elevation measurement noise is dominated by uncorrelated thermal/speckle white noise. The accuracy of the hydraulic radius estimate, *R*, is well approximated by the elevation accuracy (provided a stable estimate of the bathymetry has been obtained by prior temporal averaging). SWOT collects elevation data at high spatial resolution (azimuth spatial resolution ∼5 m, range spatial resolution ∼10 to ∼70 m) and then aggregates the results onto a series of nodes on the river centerline using a program called RiverObs (https://github.com/SWOTAlgorithms/RiverObs). The SWOT height noise level, after aggregating into centerline nodes separated by 100 m, varies between ∼10 cm for 100 m wide rivers, and ∼3.3 cm for 1 km wide rivers (Rodriguez et al., 2018).

**Figure 2:**
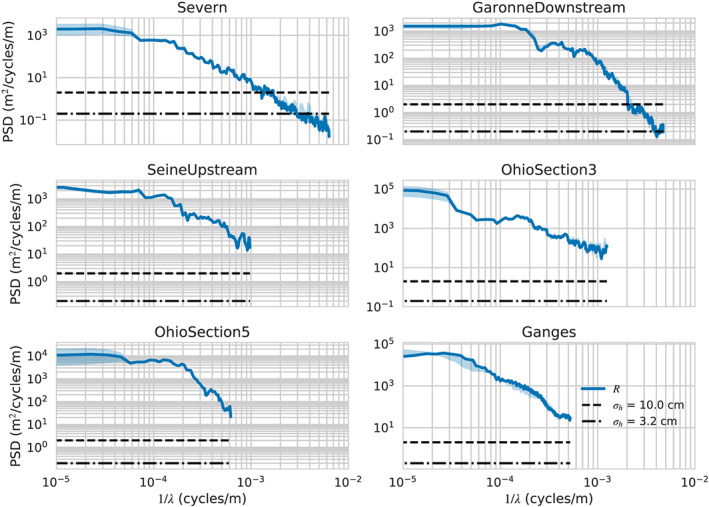
Power spectral densities (PSDs) for the hydraulic radius, *R*, and elevation measurement errors across a representative sampling of rivers. Shown are the median PSD values (solid lines) and 25–75% quantiles (shaded area) for the spectra of (blue) the hydraulic radius, *R*; (dashed line) water surface elevation measurement error sampled at 100 m with standard deviation *σ*_*h*_=10  cm. (dash‐dotted line) The same as previous but with a standard deviation *σ*_*h*_=3.3  cm. The *x* axis is the inverse of the PSD wavelength.

In Figure 2, we present PSDs for *R*, and their temporal variability, for six representative rivers spanning 4 orders of magnitude in discharge. The PSDs were estimated for the entire river segment shown in Figure 1. The PSDs show a strong power law behavior at small wavelengths, but the power law exponent flattens out significantly at longer wavelengths, consistent with long‐wavelength downstream trends. Although the spectra span significant changes in annual variability, the spectral shape for the data examined shows small variability at smaller wavelengths. Alongside the *R* PSDs, we show the PSDs for white elevation noise range for  SWOT.

The resolution criterion proposed by Chelton et al. (2018) is that, for a feature of wavelength *λ* to be resolved, the PSD level at this frequency must be greater than the noise level PSD. This means that if the hydraulic parameter were spatially filtered with a perfect low‐pass filter that allowed only signals of wavelength greater than *λ* to pass, the resulting smoothed spatial signal would have signal‐to‐noise ratio greater than 1 throughout. Conversely, all signals with smaller spatial wavelengths would have signal‐to‐noise ratios smaller than 1. Examining the intersection points of the *R* and noise PSDs, and using this criterion, we see that SWOT will resolve the hydraulic radius variability for scales of ∼500 m or better for the rivers in this study. We note that this criterion is appropriate for resolving wave‐like disturbances, such as the ones discussed by Horritt (2002) but may not be appropriate for resolving isolated features, such as jumps, in which case the scene cannot be considered to be statistically homogeneous. Thus, the resolution criterion proposed here can be considered to be an upper bound on the ability to resolve hydraulic variability. It is nonetheless a useful guide on the relative visibility of the hydraulic parameters.

The noise standard deviation for altimeter measurements is similar to the one for SWOT, but the spatial sampling will generally be much poorer (Cretaux et al., 2017). Since the noise PSD is directly proportional to the sampling interval, we would expect that altimeter sampling as fine as 10 km would result in noise PSDs that are 2 orders of magnitude higher, so the efficiency of nadir altimeters is limited to significantly longer scales of variability for *R*, and some of the smaller rivers may not be sampled adequately at all. This is consistent with the types of rivers where altimetry has been used successfully (Cretaux et al., 2017).

**Figure 3:**
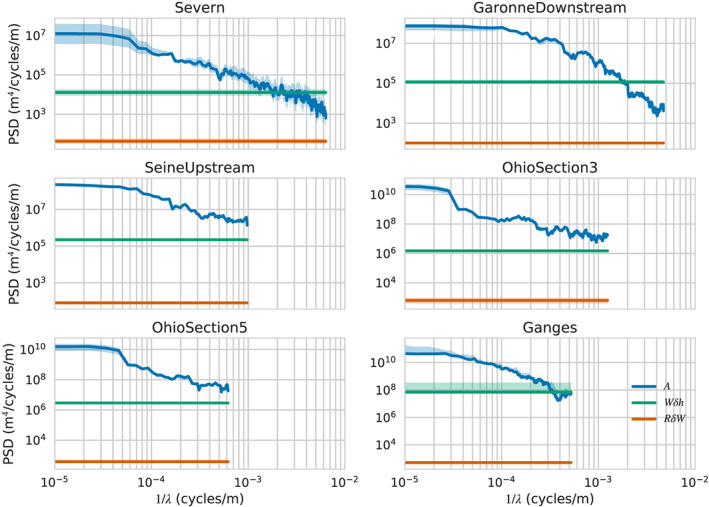
Power spectral densities (PSDs, *y* axis) for the wetted cross section, *A*, and the cross‐section measurement errors, across a representative sampling of rivers. Shown are the median PSD values (solid lines) and 25–75% quantiles (shaded area) for the spectra of (blue) the wetted cross section, *A*; (green) the spectrum of *W* *δ* *h*, assuming *σ*_*h*_=10  cm; and (orange) the spectrum of *R* *δ* *W*, assuming *σ*_*W*_=10  m.

The equivalent PSDs for the wetted cross section, *A*, are shown in Figure 3. These spectra are qualitatively similar to the ones for *R*. To get an estimate of the measurement error in *A*, we approximate *A*≈*WR*, where *W* is the river width. Since *W* errors come from imagery while *R* errors come from radar timing, they are independent. We approximate the *W* error spectra as white noise with a standard deviation of 10 m, consistent with SWOT imaging capabilities. It then follows that the *A* measurement errors can be split into two independent white noise components, *δA*≈*Wδh*+*RδW*, and they will have PSD levels of $\mathrm{var}[W]\sigma_{h}^{2}$ and $\mathrm{var}[R]\sigma_{W}^{2}$, respectively, where var[*W*] and var[*H*] represent to total variance of *W* and *R* over the river segment and $\sigma_{h}^{2}$, $\sigma_{W}^{2}$ represent measurements error variances of (0.1)^2^ and (10)^2^m^2^, respectively. In Figure 3, we show the median value of the two contributors to the noise PSD, along with the variability of these noise terms over time. Since we are dealing with wide rivers, the *Wδh* term dominates over the *RδW*, term. However, using the same noise resolution criterion that was used when discussing the *R* spectra, the accuracy that can be expected from SWOT is sufficient to resolve width variability at scales of hundreds of meters to a few kilometers.

**Figure 4:**
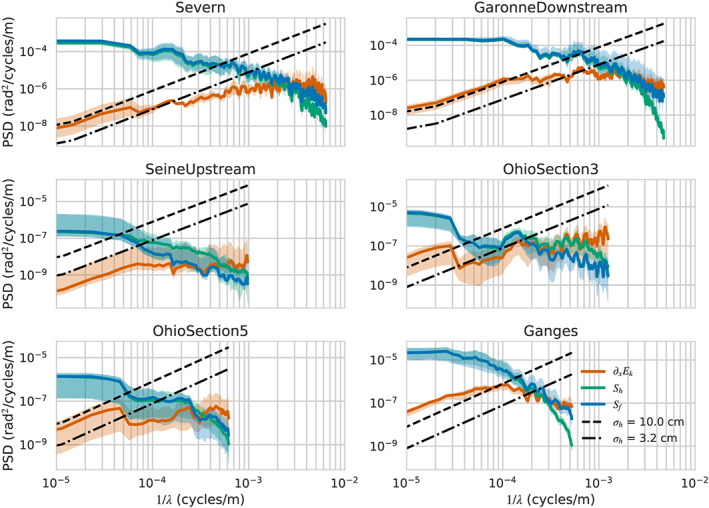
Power spectral densities (PSDs) for the friction slope, *S*_*f*_ (blue); the water surface elevation slope, *S*_*h*_ (green); the kinetic energy dissipation term, *∂*_*x*_ *E*_*k*_ (orange); and the water surface elevation slope measurement errors across a representative sampling of rivers. The water surface elevation slope error PSDs are the elevation measurement slope errors in Figure 2 multiplied by a factor of (2*π*/*λ*)^2^.

The *S*_*f*_ PSDs are shown in blue in Figure 4, and they exhibit similar power law behavior as the *R* and *A* PSDs. We also show in green the PSDs of −*∂*_*x*_ *h*, the water surface slope, which, for all rivers, follows very closely the *S*_*f*_ PSD, except at smaller scales. In two river segments, there is also some difference at 1 to 10 km scales. We hypothesize that the differences between the water surface slopes and the friction slope at small to middle scales are due to the fact that, as discussed by Hunter et al. (2007), the advective, or kinetic energy gradient, term contributes mostly at scales of hundreds of meters. To examine this hypothesis, we calculate the PSDs for *E*_*k*_=*U* ^2^/2*g* from the model data and use the fact that the PSD of the derivative of a variable is given by the PSD of that variable multiplied by *k* ^2^=(2*π*/*λ*)^2^. We show the PSD of *∂*_*x*_ *E*_*k*_ in orange in Figure 4. As expected (Hunter et al., 2007), the contribution of the kinetic energy dissipation, or advection, term is much smaller than the diffusion term at low to medium wavelengths but can dominate the water surface slope at small wavelengths. In the cases where there was disagreement between the friction and surface slopes at scales between 1 and 10 km, we see that the *∂*_*x*_ *E*_*k*_ term is also on the same order as the friction slope. (Note that the PSDs of surface slope and *∂*_*x*_ *E*_*k*_ need not add up to the PSD of *S*_*f*_ when the two spectral components are correlated, since the PSD omits phase information. The modulation of surface slope clearly influences the modulation of velocity, so we do not expect that they should be independent variables.)

To examine the hydraulic resolution capabilities for the slope terms, we also plot in Figure 4 the PSDs of the slope estimation error, which we take to be *k* ^2^ times the elevation estimation error shown in Figure 2. The *k* ^2^ factor in the derivative operator has a strong noise amplification effect, and, unlike the previous cases, we see that the measurement errors in the slope impose significant limitations on the hydraulic observability of the slope terms. The resolution wavelength lies between about ∼1 km, for the steeper rivers, and ∼10 km (or greater) for the low‐slope rivers. We also see that the *∂*_*x*_ *E*_*k*_ term is not visible, except at very long wavelengths, when its contribution is insignificant relative to the water surface slope. This observation explains the great sensitivity to errors for for this term found by Garambois and Monnier (2015) and justifies the use of the change kinematic diffusion wave approximation in studies for the inversion of discharge from SWOT‐like data (Durand et al., 2010, 2014; Garambois & Monnier, 2015; Yoon et al., 2016).

In Table 2, we present the wavelength at which the slope noise spectra cross the *S*_*f*_ PSD for all of the rivers in this study. As can be seen, the hydraulic *S*_*f*_ resolution varies significantly between streams and, due to the power law behavior, between noise levels. SWOT can resolve small, high‐slope streams at high resolution (e.g., Severn) but may need significant averaging for some low‐slope rivers (e.g., Ohio sections 4 and 5). Although these resolution numbers are only indicative of the reach size that should be used, they generally suggest reach lengths that can be significantly longer or shorter than those based on other considerations (Frasson et al., 2017b), and this spectral criterion should enter considerations for the definition of observable SWOT reaches.

**Table 2:** Crossing Wavelengths (*Hydraulic Resolution Wavelength*) for the Intersection of the *S*_*f*_ PSD and the Slope Noise PSD, for Two Levels of Height Noise Characteristic of the Range of the SWOT Performance

	*σ*_*h*_=10 cm	*σ*_*h*_= 3 cm
River name	Resolution (km)	Resolution (km)
Severn	2.9	1.1
Seine upstream	29.1	14.8
Garonne upstream	0.9	0.5
Sacramento upstream	1.6	0.9
Sacramento downstream	7.6	3.1
Seine downstream	30.5	16.2
Arial Khan	24.8	16.7
Garonne downstream	2.3	1.1
Kanawha	17.1	10.4
Kushiyara	17.3	0.2
Cumberland	17.4	8.8
Ohio section 1	54.0	19.5
Ohio section 7	31.6	14.1
Ohio section 2	30.1	17.4
Ohio section 3	26.2	18.2
Ohio section 4	232.5	27.3
Ohio section 5	28.4	17.9
Ganges	8.5	4.7
Mississippi upstream	6.5	2.9
Mississippi intermediate	7.0	3.1
Ohio section 8	15.0	7.9
Mississippi downstream	6.4	3.3
Padma	16.5	9.6

As discussed above, the altimeter noise PSD will be several orders of magnitude greater than that of SWOT. This will limit the slope hydraulic resolution to long wavelengths, which is consistent with the altimeter slope estimates that have been presented in the literature (e.g., Birkett et al., 2002; Garambois et al., 2017).

In addition to the terms discussed above, two additional terms contribute to *S*_*f*_ in equation (4): the acceleration, *∂*_*t*_ *U*, and the lateral discharge proportional to *q*. For the available model runs and temporal sampling, we found that, for the cases studied, the acceleration term was significantly smaller than the other terms, although it could have a significant contribution for isolated events. Since this behavior is neither homogeneous nor stationary, it does not lend itself to analysis by the PSD: On overage the effect was too weak to be observable, but these are probably not cases were it is of interest. The daily sampling used in the data set may also have underestimated the magnitude of the acceleration  term.

The model cases we studied did not have significant contribution from lateral inflows for each reach, and we cannot assess the spatial scales at which this term would be observable by SWOT. The validity of the assumption that lateral discharge can be neglected over a reach may not be easily tested because the currently available relevant data have too much uncertainty to validate this assumption (P. Bates, private communication, July 2019). For small rivers, for example, Silliman and Booth (1993) found groundwater‐surface water interactions not to be negligible. Durand et al. (2014) found that lateral flows could be estimated from elevation data and imagery the Severn river, including a flood event, albeit at the cost of significant increase in estimated parameter errors. Even for larger rivers (e.g., Stewart et al., 1999) nontributary hillslope runoff has been hypothesized to lead to nonnegligible changes in discharge at scales of tens of kilometers. Recently, Nickles (2019) has studied the impact of lateral discharge around a storm event on SWOT discharge retrieval algorithms and found that, when it was neglected, large errors could occur, but that even coarse estimates would result in significant improvement of estimates of total discharge. We leave the spatial scale of the hydraulic visibility of lateral discharge for future work that includes model cases with realistic runoff contributions. We note below how the presence of lateral inflows within a reach impacts our results.

## 4 Analytical Computations

This section shows the effect of spatial averaging on reach‐averaged Saint Venant equation (i.e., the right‐hand side of equation 4), introduces a variability index into the typical power law formulation of *S*_*f*_ (i.e., the left‐hand side of equation 4), and finally relates that variability index to hydraulic parameter dispersion.

### 4.1 The Reach‐Averaged Saint Venant Equation

The hydraulic visibility results of the previous section show that the hydraulic variables must be smoothed for distances that can be on the order of 10 km before they have a signal‐to‐noise ratio greater than 1. It is not evident that these smoothed variables will satisfy the Saint Venant equations, and we derive here their dynamic equations. We decompose each hydraulic variable, for example, *U*, into a smoothed component (denoted by, e.g., $\bar{U}$), obtained by convolving the variable with a smoothing filter (see details in section S2 of the supporting information) and a fluctuating component (denoted by, e.g., *δU*), representing the within‐reach variability, for example, $U=\bar{U}+\delta U$. Applying the reach‐averaging filter to the Saint Venant equations and using the fact that averaging and differentiation can be interchanged (see supporting information), we obtain

(5) $$\bar{q}=\frac{\partial \bar{A}}{\partial t}+\frac{\partial \bar{Q}}{\partial x}$$

(6) $$\bar{S_{f}}=-\frac{\partial \bar{h}}{\partial x}-\beta \frac{\partial }{\partial x}\bar{\frac{U^{2}}{2g}}-\frac{1}{g}\left[\frac{\partial \bar{U}}{\partial t}+\bar{\left[\frac{Uq}{A}\right]}\right]$$

Aside from two nonlinear terms, these equations involve only the reach‐averaged hydraulic variables. Using the fact that the reach average of the fluctuating terms vanishes, one can rewrite the momentum equation in terms of the reach‐averaged quantities and the quadratic moments of the fluctuations

(7) $$\bar{S_{f}}=-\frac{\partial \bar{h}}{\partial x}-\beta \frac{\partial }{\partial x}\frac{\beta_{R}{\bar{U}}^{2}}{2g}-\frac{1}{g}\left[\frac{\partial \bar{U}}{\partial t}+\beta_{q}\frac{\bar{U}\bar{q}}{\bar{A}}\right]$$

(8) $$\beta_{R}=\left[1+\frac{\bar{\delta U^{2}}}{{\bar{U}}^{2}}\right]$$

(9) $$\beta_{q}\approx 1+2\frac{\bar{\delta A^{2}}}{{\bar{A}}^{2}}+\frac{\bar{\delta U\delta q}}{\bar{U}\bar{q}}-\frac{\bar{\delta A\delta q}}{\bar{A}\bar{q}}$$

In general, we do not expect the *fluctuations* of lateral discharge to be correlated with the *fluctuations* of velocity or cross section, so we approximate $\beta_{q}\approx 1+2\bar{\delta A^{2}}/{\bar{A}}^{2}$.

If we assume that *Q* is approximately conserved over the reach, $\delta Q/\bar{Q}\ll 1$ and one has that the fluctuations in *A* and *U*, which need not be small, must satisfy

(10) $$\frac{\delta U}{\bar{U}}+\frac{\delta A}{\bar{A}}\approx \frac{\delta Q}{\bar{Q}}\approx 0$$

from which follows that

(11) $$\frac{\bar{\delta U^{2}}}{{\bar{U}}^{2}}\approx \frac{\bar{\delta A^{2}}}{{\bar{A}}^{2}}$$

$\beta_{R}\approx 1+\bar{\delta A^{2}}/{\bar{A}}^{2}>1$ and *β*_*q*_≈2*β*_*R*_−1. If *q* cannot be neglected over the reach, the simple relationship between cross section and velocity fluctuations will not apply, and the reach‐averaged Saint Venant equations will depend on two parameters, *β*_*R*_ and *β*_*q*_, rather than a single parameter *β*_*R*_.

**Figure 5:**
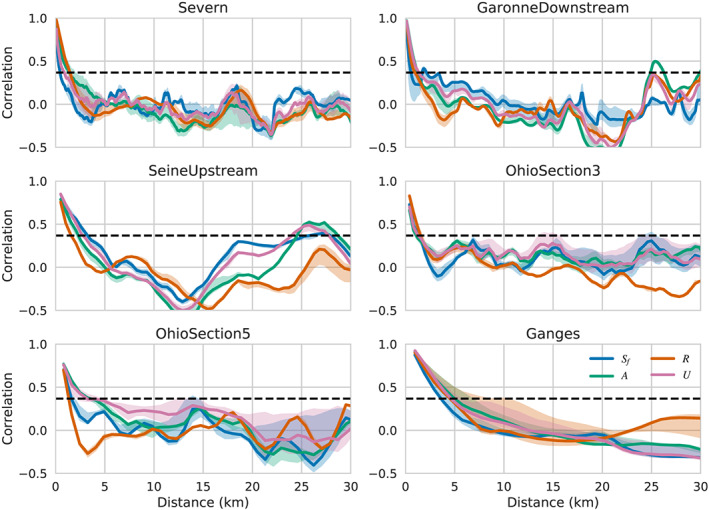
Spatial correlation functions for friction slope, *S*_*f*_ (blue); wetted cross section, *A* (green); hydraulic radius, *R* (orange); and river velocity, *U*, as a function of separation for a representative set of rivers. The solid lines represent the median value of the correlation over all times, while the shaded areas represent the variation between the 25% and 75% quartiles. The dashed black line represents the 1/*e* value used to derive the correlation distances given in Table 3.

Equations  7 and 5 are almost of the form we want but do not yet form a closed system since they involve location‐dependent fluctuation terms, through the *β*_*R*_ and *β*_*q*_, whose dynamics are not yet specified. In analogy with the assumptions behind the Boussinesq *β*, we hypothesize that if the reach length is long enough to contain many independent realizations of the fluctuations, one can replace the fluctuating terms by stationary statistical averages that characterize hydraulic parameter statistics (Moody & Troutman, 2002) appropriate for this river type and which may be available from other remote sensing data (e.g., Allen & Pavelsky, 2018; Allen et al., 2018). To estimate the minimum averaging length required to reach a stable value of the fluctuating terms, we assume that it must be larger than the parameter correlation length. In Figure 5, we show representative estimates of the correlation function, *C*_*p*_(*d*), defined for a generic hydraulic parameter *p* as

(12) $$C_{p}(d)=1-\frac{\left\langle {\left(p(x)-p(x+d)\right)}^{2}\right\rangle }{2\left\langle p{\left(x\right)}^{2}\right\rangle }$$

where *d* is the downstream separation between two points in the reach.

In order to minimize nonhomogeneous long‐term secular trends, we remove a second‐order polynomial over the entire river length, prior to using this formula. We estimate, *L*_*Cp*_, the correlation distance for parameter *p*, by solving *C*_*p*_(*L*_*Cp*_)=1/*e*; that is, by solving for the intersection of the median value of the correlation functions in Figure 5 with the black dashed line. The resulting correlation values for *S*_*f*_, *A*, *R*, and *U* are given in Table 3. Comparing with Table 2, we see that, in general, the parameter correlation length is smaller than the resolvable reach, so that we expect that *β*_*R*_ over the reach will be approximately constant, and its spatial derivatives can be neglected. With this approximation we derive the reach‐averaged momentum Saint Venant equation in terms of the reach‐averaged hydraulic parameters:

(13) $$\bar{S_{f}}=-\frac{\partial \bar{h}}{\partial x}-\beta \beta_{R}\frac{\partial }{\partial x}\frac{{\bar{U}}^{2}}{2g}-\frac{1}{g}\left[\frac{\partial \bar{U}}{\partial t}+\left(2\beta_{R}-1\right)\frac{\bar{U}\bar{q}}{\bar{A}}\right]$$

**Table 3:** Hydraulic Parameter Correlation Distance, *L*_*C*_, for the Rivers in This Study

	$L_{CS_{f}}$	*L*_*CA*_	*L*_*CR*_	*L*_*CW*_	*L*_*Z*_		
River name	(km)	(km)	(km)	(km)	(km)	med(*L*_*f*_)	mad(*L*_*f*_)
Severn	0.5	1.4	1.5	1.3	1.6	1.7	0.8
Seine upstream	3.2	2.3	1.6	1.0	2.0	264.6	231.1
Garonne upstream	0.4	0.5	0.5	0.5	5.9	0.4	0.2
Sacramento upstream	0.5	0.6	0.5	0.5	0.5	1.8	0.5
Sacramento downstream	0.5	0.5	0.7	1.5	0.6	17.9	12.3
Seine downstream	1.0	5.8	5.6	0.9	1.7	197.9	142.0
Arial Khan	5.3	5.6	5.2	4.6	5.0	132.9	155.6
Garonne downstream	0.6	1.2	1.0	0.9	1.6	5.0	3.1
Kanawha	5.5	7.8	7.1	1.1	2.1	110.8	112.3
Kushiyara	9.3	12.2	9.5	12.4	8.1	71.6	85.4
Cumberland	2.4	2.2	2.5	2.3	1.9	103.5	103.6
Ohio section 1	2.7	3.5	0.8	0.9	2.2	265.7	332.3
Ohio section 7	2.2	3.6	2.7	3.0	2.5	217.9	255.4
Ohio section 2	2.1	2.3	3.1	1.5	2.2	156.2	160.3
Ohio section 3	1.3	1.1	1.5	1.4	1.6	147.2	155.6
Ohio section 4	2.3	1.1	1.2	0.9	1.1	625.1	785.9
Ohio section 5	1.6	3.5	1.3	1.3	1.4	134.0	125.0
Ganges	3.6	5.0	4.5	5.3	5.1	35.6	20.3
Mississippi upstream	1.5	2.1	1.5	1.3	1.5	19.4	6.4
Mississippi intermediate	1.1	1.5	1.1	1.1	1.2	16.6	4.8
Ohio section 8	2.6	2.5	2.6	2.9	3.0	77.9	37.9
Mississippi downstream	1.7	2.1	2.3	2.7	2.4	29.2	7.4
Padma	4.0	4.4	2.8	3.3	2.9	74.2	76.1

*Note*. The correlation distance is defined as the intersection of the correlation coefficient curve with the 1/*e* line, as shown in Figure 5. Also shown are the median and median absolute deviations of *L*_*f*_ over all reaches.

This equation and equation  5 are formally identical to the Saint Venant equations aside from the $\beta_{R}\approx 1+\bar{\delta A^{2}}/{\bar{A}}^{2}$ terms, which shows that the main impact of within‐reach variability can be attributed to the fractional variability of the wetted cross section. In the limit where this variability can be ignored, *β*_*R*_→1, and the exact Saint Venant equations are recovered. The previous two equations do not yet form a closed system of equations due to the presence of $\bar{S_{f}}$ and $\bar{Q}$, which are not yet expressed in terms of the reach‐averaged variables: That is the subject of section 4.2.

It is instructive to consider the case when the reach‐averaging function corresponds to uniform weighting between upstream, *x*_*u*_, and downstream, *x*_*d*_, locations. If we ignore the acceleration and lateral inflow terms, consistent with steady gradually varied flow, and use the results in the supporting information, the reach‐averaged momentum equation becomes

(14) $$h(x_{u})+\beta \frac{U^{2}(x_{u})}{2g}=h(x_{d})+\beta \frac{U^{2}(x_{d})}{2g}-\bar{S_{f}}\left|x_{d}-x_{u}\right|$$

which is the usual energy conservation equation and contains the *instantaneous* (not reach averaged) values for the water surface elevation and velocity. The only difference with the usual equation is that the *average* friction slope appears, which depends on the *average* Manning friction coefficient, to be derived in the next section. Since these terms are usually *calibrated* in models based on cross sections, such as HEC‐RAS, these calibrated models may in fact implicitly implement the reach‐averaged Saint Venant equations derived here. These results show that the result of reach averaging is to modify the *S*_*f*_ term that accounts for dissipative energy loss. This energy loss is usually thought to be due to wall friction but, in the reach‐averaged cases, can also include hydraulic variability, for example, to include kinetic energy loss due to channel variations in a riffle and pool system which is not directly observable between two cross sections.

### 4.2 Introducing a Variability Index Into the Friction Slope

In the previous section, we saw that the reach‐averaged friction coefficient, $\bar{S_{f}}$, dictates the energy lost over a reach. In the present section, we derive a relationship between $\bar{S_{f}}$ and the mean parameters in the reach and show that it has the identical functional form as the relationship at a point, with local parameters replaced by the reach‐averaged mean, except for one additional term which we call a “variation index.” This variation index can be either included in the model for *S*_*f*_ explicitly or used to obtain an effective friction coefficient. In the following section, we derive the relationship between the increases in the friction coefficient and the spatial variability of the hydraulic parameters.

Rather than consider the friction coefficient directly, since it can fluctuate strongly within the reach, we consider the discharge at a point, *Q*(*x*), which is usually nearly constant over a suitably defined reach. We relate it to the friction slope through $Q(x)=K(x)S_{f}^{1/2}(x)$, where K is the conveyance (Dingman, 2009), which is inversely proportional to a friction parameter, *ρ*, and proportional to the product of river hydraulic parameters, raised to some power. (Table 4 presents the parameters and exponents for common parametrizations of the conveyance Dingman, 2009). This allows us to write the discharge as a function of the form

(15) $$Q(x,p)=\overset{N_{p}}{\underset{i=1}{\prod }}p_{i}^{\alpha_{i}}(x)$$

where *p*_*i*_ is the *i*th river hydraulic parameter and *α*_*i*_ is the corresponding exponent, and we take the friction parameter, *ρ*, to be the *i*=*N*_*p*_ parameter.

**Table 4:** Parameters and Exponents *α* in the Manning Equation (Third Column), the Darcy‐Weisbach Equation, and Two Versions of Manning's Equation That Apply When the Characteristic Width Is Much Larger Than the Characteristic Depth

Symbol	Parameter	Manning	Manning shallow 1	Manning shallow 2	Darcy‐Weisbach	Chézy
*N*_*p*_		4	4	4	3	4
*P*	Wetted perimeter	0	0	0	0	0
*A*	Cross‐section area	1	5/3	0	0	0
*W*	Width	0	−2/3	1	0	1
*S*_*f*_	Friction slope	1/2	1/2	1/2	1/2	1/2
*R*=*A*/*P*	Hydraulic radius	2/3	0	5/3	5/2	3/2
*ρ*	Friction	*n*	*n*	*n*	$\sqrt{\frac{8f_{D}}{\pi^{2}g}}$	$\frac{\Omega }{g^{1/2}}$

*Note*. The Manning coefficient is denoted by *n* (dimensional and must be corrected for units), while *f*_*D*_ is the Darcy friction factor, Ω is the Chézy flow resistance, and *g* is the acceleration of gravity.

To derive the reach‐averaged discharge equation (detailed derivation steps are given in section S3 of the supporting information), take the logarithm of equation 15 and average the result over the reach to obtain

(16) $$\bar{\log(Q)}=\overset{N_{p}}{\underset{i=1}{\sum }}\alpha_{i}\bar{\log(p_{i})}$$

Because of the nonlinearity of the logarithm, $\bar{\log p_{i}}\neq \log\bar{p_{i}}$ in general, unless *p*_*i*_ is constant. In fact, using Jensen's inequality (Jensen, 1906), one has that $\bar{\log p_{i}}\leq \log\bar{p_{i}}$. We characterize this difference through a positive indefinite variability index *κ*_*i*_ defined by

(17) $$\bar{\log p_{i}}=\log\bar{p_{i}}-\log(1+\kappa_{i})$$

Using this equation, one can write the reach‐averaged equation as

(18) $$\log\bar{Q}=\overset{N_{p}-1}{\underset{i=1}{\sum }}\alpha_{i}\log\bar{p_{i}}-\log\tilde{\rho }$$

(19) $$\tilde{\rho }=\left[\frac{1}{\left(1+\kappa_{Q}\right)}\overset{N_{p}}{\underset{i=1}{\prod }}{\left(1+\kappa_{i}\right)}^{\alpha_{i}}\right]\bar{\rho }\equiv \left(1+\kappa_{T}\right)\bar{\rho }$$

where *κ*_*Q*_ is defined analogously to *κ*_*i*_, and we define the total variability index as *κ*_*T*_. Taking the exponential, we find the final form for the reach‐averaged discharged equation to be

(20) $$\bar{Q}=\frac{1}{\tilde{\rho }}\overset{N_{p}-1}{\underset{i=1}{\prod }}{\bar{p_{i}}}^{\alpha_{i}}$$

which is identical in form with the point discharge equation but with a different value of the friction coefficient. This equation can be inverted for $\bar{S_{f}}$ in terms of $\bar{Q}$, the reach‐averaged hydraulic variables, and the rescaled Manning coefficient, to complete the reach‐averaged Saint Venant equations as function of reach‐averaged variables.

What can be said about the new friction coefficient? First, from Jensen's inequality, we know that *κ*_*i*_ ≥ 0 for all the parameters. There is a term in the renormalized friction coefficient which is inversely proportional to the discharge variability, due to lateral flows, which will appear to reduce the friction coefficient. If we restrict ourselves to reaches where lateral flows are negligible, so that the discharge is (approximately) conserved, then the internal variability of the river parameters will always cause the renormalized friction coefficient to increase. This is analogous to accounting for unresolved turbulence in the Navier‐Stokes equation by rescaling the diffusion parameter to values significantly greater than the molecular diffusion term. In the Saint Venant equation, the friction coefficient plays the function of parametrizing the stress at the riverbed, which reduces the kinetic energy. To keep the discharge constant in the less variable river described by the reach‐averaged hydraulic variables, a greater effective friction is required to slow down the flow and satisfy energy, equation (14).

Additional insight into the source of *κ*_*T*_ can be gained if we assume that the river parameters are sampled at a spatial scale small enough so that they vary linearly between samples. In that case, reach averaging (equation (S6) in the supporting information) can be replaced by

(21) $$\bar{p_{i}}=\frac{\sum_{k=1}^{N_{x}}p_{i}(x_{k})}{N_{x}}$$

where *x*_*k*_ are the sample downstream coordinates and *N*_*x*_ are the number of sampling locations. Then, it follows that

(22) $$\bar{\log p_{i}}=\log\left({\left[\overset{N_{x}}{\underset{k=1}{\prod }}p_{i}(x_{k})\right]}^{1/N_{x}}\right)$$

(23) $$=\log\hat{p_{i}}$$

where $\hat{p_{i}}$ is the *geometric* mean of *p*_*i*_ over the reach. From equation (17), it then follows that the *κ*_*i*_ must be of the form

(24) $$\kappa_{i}=\frac{\bar{p_{i}}}{\hat{p_{i}}}-1$$

that is, *κ*_*i*_ is a measure of the deviation of the *arithmetic* mean from the *geometric* mean. Therefore, *κ*_*i*_ is a measure of the presence of large tails in the distribution of the parameter. The relationship between geometric and arithmetic means has previously been used to derive the reach‐averaged hydraulic relations by Harman et al. (2008), who also examine the relationship in logarithmic space. We note that for variables that are lognormal, the geometric mean corresponds to the median value (Forbes et al., 2011), so the reach‐averaged Manning equation relates the medians of the hydraulic parameters for lognormal variables.

### 4.3 Relating the Variability Index to Variance of Hydraulic Parameters

If full spatial sampling of the parameters is available within a reach, equations 17–20 are exact equations that can be used to predict the effective Manning friction coefficient. However, the main reason to go to reach‐averaged equations is that the within‐reach parameter samples often are not known precisely from remote sensing (as shown in the previous section) a priori, although statistics about the variability may be available. In this section, we seek to estimate *κ*_*T*_ knowing the parameter variances and making assumptions about their distribution.

To examine the impact of within‐reach fluctuations to the variability index, define

(25) $$\epsilon_{i}(x)=\frac{p_{i}(x)-\bar{p_{i}}}{\bar{p_{i}}}$$

(26) $$\zeta_{i}(x)=\log\left(1+\epsilon_{i}(x)\right)$$

We note that if the parameters are normally distributed about their mean, *ϵ*_*i*_ will be normally distributed with zero mean. If, as seems more likely given observations by Allen et al. (2018), Frasson et al. (2019), Moody and Troutman (2002), they are lognormal, then *ζ*_*i*_ will be normally distributed, with mean given by

(27) $$\bar{\zeta_{i}}=-\log\left(1+\kappa_{i}\right)$$

where we used equation 17.

To establish a relationship between *κ*_*T*_ and the parameter variabilities for weak fluctuations, we note, using equation 19, that

(28) $$-\log\left(1+\kappa_{T}\right)=\overset{N_{p}}{\underset{i=1}{\sum }}\alpha_{i}\bar{\zeta_{i}}+\log\left(1+\kappa_{Q}\right)$$

Assuming that the parameters follow the lognormal distribution, one can express $\bar{\zeta_{i}}=-\log(1+\kappa_{i})$ in terms of $\bar{\epsilon_{i}^{2}}$ (Forbes et al., 2011).

(29) $$-\log(1+\kappa_{i})=\log\left[\frac{1}{\sqrt{1+\bar{\epsilon_{i}^{2}}}}\right]$$

Assuming that *Q* is also lognormally distributed, so that a similar relation applies to *κ*_*Q*_, we can replace these results into equation (28), combine the logarithms, and take the exponential, to solve for *κ*_*T*_

(30) $$\kappa_{T}=\frac{\overset{N_{p}}{\underset{i=1}{\prod }}{\left(1+\bar{\epsilon_{i}^{2}}\right)}^{\alpha_{i}/2}}{\sqrt{1+\bar{\epsilon_{Q}^{2}}}}-1$$

This result is exact, independent of the magnitude of the within‐reach fluctuations, as long as the parameters follow the lognormal distribution. In the weak fluctuation limit, $\bar{\epsilon_{i}^{2}}\ll 1$, this becomes

(31) $$\kappa_{T}\approx \frac{1}{2}\underset{i}{\sum }\alpha_{i}\bar{\epsilon_{i}^{2}}-\frac{1}{2}\bar{\epsilon_{Q}^{2}}$$

As we will see in the discussion of model results below, the weak fluctuation limit seems to apply when the normalized variabilities (or *κ*_*T*_) are on the order of 0.3–0.5 or smaller. These weak modulation results are also valid independent of any assumptions regarding the statistical distribution of the parameters, as long as moments of the distribution higher than 2 are neglected, as can be seen by expanding equations (26) and (28) to second order in *ϵ*_*i*_. We note that the effects of lateral discharge are through the term $-\bar{\epsilon_{Q}^{2}}/2$, which leads to a *decrease* of the effective roughness coefficient. This leads to an *increase* of the effective friction slope, leading to an increase in the effective velocity, needed to accommodate the increase in discharge.

Although the previous expressions involve the parameter variances, they do not involve the covariances. This does not mean that the parameters are independent: In log‐space their fluctuations are linearly dependent, as the following analysis shows. Defining $\eta_{i}(x)=\zeta_{i}(x)-\bar{\zeta_{i}}$, we can write the following equation which holds at each point *x*:

(32) $$\log Q(x)=\log\bar{Q}+F(x)$$

(33) $$F(x)=\overset{N_{p}}{\underset{i=1}{\sum }}\alpha_{i}\eta_{i}(x)$$

where $\bar{Q}$ is given by equation (20). If we restrict ourselves to reaches with constant discharge, $\log Q(x)=\log\bar{Q}$, and the fluctuation term *F*(*x*) vanishes everywhere; that is, when discharge is conserved, the fluctuations of the log parameters must lie on an (*N*_*p*_−1)‐dimensional hyperplane defined by

(34) $$\sum \alpha_{i}\eta_{i}(x)=0$$

Notice that this result is exact for conserved discharge, given known within‐reach fluctuations, and no statistical assumptions have been made. In contrast, there is no equivalent simple relationship for the *ϵ*_*i*_, and they vary in complicated nonlinear fashion, as we will see in the following sections. When discharge is not conserved, the parameters at each *x* will still vary on a hyperplane, but the hyperplane will not intersect the origin, and the point of intersection will depend on the local lateral discharge.

The fact that the log parameters have to restrict themselves to a hyperplane does allow us to make predictions about the covariance of the parameters. In principle, this will allow the prediction of the variability of an unobserved parameter if estimates of the other parameter variabilities are known. Since it is incidental to the main argument, but useful in trying to parametrize the effective Manning coefficient given limited observations, we present these results in section S4 of the supporting information.

## 5 Results

### 5.1 Spatial Averaging the Low‐Froude Flow Law

As noted above, many studies (e.g., Durand et al., 2016) use simple flow laws such as Manning's equation to relate surface slope directly to discharge. As noted by Garambois and Monnier (2015), simple flow laws used in this way implicitly assume that the Froude number is less than approximately 0.3. Under the low‐Froude approximation, the surface slope *S* is equal to *S*_*f*_. In this section, we examine the effect of spatial variability on a simple form of Manning's equation used as a flow law under the low‐Froude approximation. Assuming a reach with steady, mass‐conserved flow and a large width‐to‐depth ratio, then at any location within the reach

(35) $$Q=\frac{1}{n}H^{5/3}WS^{1/2}$$

where *W* is river width and *H* is the flow depth. Assuming no spatial variability in surface slope or friction coefficient, but that width and depth co‐vary, then using equations (19) and (20) gives

(36) $$Q=\frac{1}{n}{\bar{H}}^{5/3}\bar{W}S^{1/2}{\left(1+\kappa_{T}\right)}^{-1}$$

where the $\bar{H}$ and $\bar{W}$ indicate reach‐averaged depth and width, respectively. Using the weak fluctuation limit, equation (31) then allows a form of Manning's equation that is explicitly dependent on the width and depth variabilities:

(37) $$Q=\frac{1}{n}{\bar{H}}^{5/3}\bar{W}S^{1/2}{\left(1+\frac{1}{2}\frac{\sigma_{W}^{2}}{{\bar{W}}^{2}}+\frac{5}{6}\frac{\sigma_{H}^{2}}{{\bar{H}}^{2}}\right)}^{-1}$$

where *σ*_*H*_ and *σ*_*W*_ are the spatial variability in depth and width, respectively. A similar form could be obtained using the lognormal assumption (equation (30)).

To further illustrate this idea, we present a simple Monte Carlo example. Assume a reach using *n*=0.04, *Q*=50, *S*=30  cm/km, all spatially constant; assume an average width of 100 m. If there were no spatial variability, then the average depth would be 1.09 m, and the Froude number would be 0.14: well within the low‐Froude approximation. Then assume that width spatial variations follow a lognormal distribution characterized by standard deviation *σ*_*W*_. For this example, we generated a random set of widths within the reach (*N*=10,000); depths were then computed by solving equation (35), and $\bar{H}$ and *σ*_*H*_ were computed across the resulting depth values. These random realizations can be considered spatial variations or cross sections.

**Figure 6:**
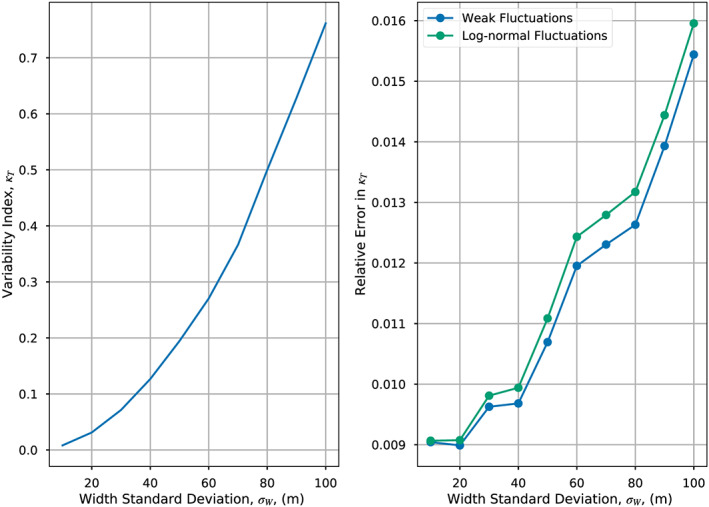
(left) True *κ*_*T*_ as a function of river width standard deviation from Monte Carlo experiment described in section 5.1. (right) Relative errors in the estimate of *κ*_*T*_ in the Monte Carlo experiment assuming weak (blue) or lognormal (green) width fluctuations.

Figure 6 shows the resulting values of *κ*_*T*_ for different values of *σ*_*W*_; we compare equation (36) (which is essentially the “true” value of *κ*_*T*_) with the estimate from the weak fluctuation estimate given by equation (37). For relatively low values of *σ*_*W*_ (<30 m, compared with a mean value of 100 m), *κ*_*T*_ is less than 0.07. However, as *σ*_*W*_ increases up to 100 m (i.e., 100% of the mean value), *κ*_*T*_ reaches a maximum value of 0.76. This underscores the importance of parameter variabilities. If *κ*_*T*_=0.76, the final term on the right‐hand side of equation (36) is 0.56; ignoring spatial variability in this context would lead to an error in discharge of 54%. Figure 6 also shows the errors in approximating *κ*_*T*_ using the weak fluctuation theory and the lognormal approximations: In both cases, errors increase with *σ*_*W*_ but are always less than 1.5%. This simplistic example has highlighted the importance of spatial variability in this application and the analytical dependence of the adapted flow law on spatial variance of the hydraulic parameters.

**Figure 7:**
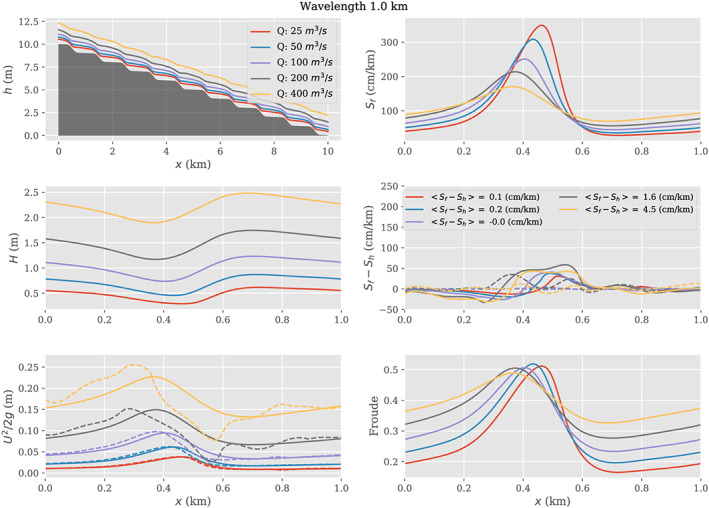
Riffle and pool toy model results for a riffle/pool wavelength of 1 km. (Upper left panel) Ripple and pool bed elevation (gray shading) over 10 km downstream distance, together with water surface elevation solutions from equation (38) for discharge values of 25 m^3^/s (red), 50 m^3^/s (blue), 100 m^3^/s (purple), 200 m^3^/s (gray), and 400 m^3^/s (yellow) (color conventions are retained throughout). (Middle left) Water depth over one wavelength as a function of discharge. (Lower left) Kinetic energy head, *U* ^2^/2*g*, as a function of discharge for the exact solution (solid lines) and a solution based on using the water surface elevation slope, *S*_*h*_, to calculate *U*. (Upper right) Friction slope, *S*_*f*_, as a function of discharge. (Middle right) Kinetic energy dissipation, *∂*_*x*_ *U* ^2^/2*g*, equal to the difference between friction and water level slopes from the exact solution (solid lines) and obtained by iterating the Saint Venant equation given the observables (dashed lines). In the legend, the residual values averaged over a wavelength as reported. (Lower right) Froude number, *F*_*r*_, as a function of discharge.

**Figure 8:**
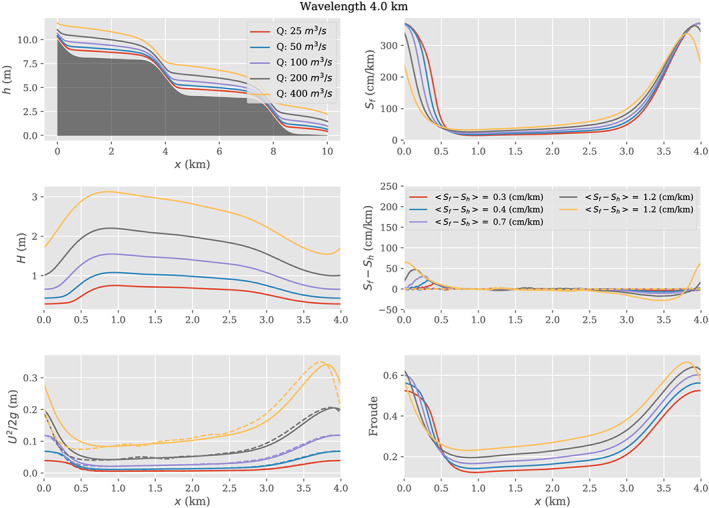
As in Figure 7 but for a riffle/pool wavelength of 4 km.

### 5.2 Riffle and Pool Example

We illustrate the reach‐averaged results using a simulated periodic riffle and pool sequence, illustrated in Figures (7) and (8), which can be solved to arbitrary precision using the steady gradually varying flow equation (Chaudhry, 2008)

(38) $$\frac{\partial H}{\partial x}=\frac{-\partial_{x}Z_{0}-S_{f}}{1-F_{r}^{2}}$$

where *H* is the flow depth, *Z*_0_ is the bed bathymetry, and *F*_*r*_ is the Froude number given by $F_{r}=U/\sqrt{gH}=Q/\left(WH\sqrt{gH}\right)$. We assume a simple rectangular channel of width *W* and constant Manning coefficient *n*, so that, from the Manning equation, the friction slope is $S_{f}={\left(nQ/WH^{5/3}\right)}^{2}$. Section S5 of the supporting information gives the equations used for modeling the bathymetry, which has a variable periodicity *L*, and reduces to a bed with constant slope *S*_0_, after reach averaging. *S*_0_ is independent of the riffle‐pool period and the pool slope *S*_*p*_ which is an order of magnitude smaller than the mean slope. By varying *L*, we obtain a set of very different bathymetries that would be indistinguishable to an instrument, like SWOT, that could only resolve reach lengths of 10 km. To use realistic parameters, we use values characteristic of the Garonne River, near Toulouse, and list them in Table 5. The discharge is varied between 25 and 400 m^3^/s, consistent with climatological observations of the Garonne near Toulouse. Equation (38) is solved for *H* using a fourth‐order Runge‐Kutta solver (Chaudhry, 2008) assuming that the downstream depth is given by the normal depth, given the discharge.

**Table 5:** Parameters Used in the Riffle and Pool Model

Parameter	Value
River width *W*	100 m
Manning coefficient *n*	0.03 s/m^1/3^
Mean slope *S*_0_	1 m/km
Pool slope *S*_*p*_	0.1 m/km
Maximum slope	∼4*S*_0_
Riffle‐pool wavelengths *L*	0.5, 1, 4, and 10 km

**Figure 9:**
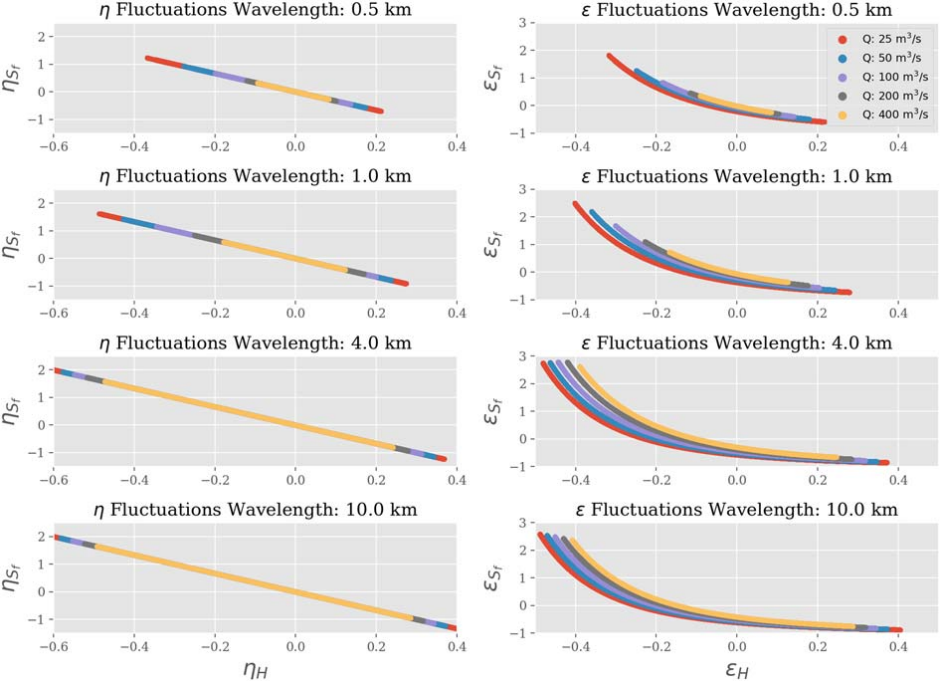
(Left column) Joint fluctuations of the log variables, *η*_*Sf*_ *η*_*H*_, for the riffle and pool model as a function of discharge (color convention as in Figure 7) and riffle/pool wavelength (from 0.5 to 10 km). (Right column) Same as left column but for the linear variables *ϵ*_*Sf*_, *ϵ*_*H*_.

This model has only two variables that fluctuate within the reach: *H* and *S*_*f*_. In Figure 9 we show that the fluctuations of the log parameters from the numerical solution of equation 38, *η*_*H*_ and $\eta_{S_{f}}$, always lie along the identical line, within numerical error, as predicted by equation 34. This result is independent of the value of the discharge *Q* or the riffle‐pool wavelength *L*. We note that the range of the *η*'s (or *ϵ*'s) increases with increasing pool wavelength because for short wavelengths (e.g., Figure 7), the riffle sequences are short and the depth does not have time to adjust to the slope increases, whereas for longer wavelengths

(e.g., Figure 8) the longer riffle sequences lead to greater depth modulations. A quantitative discussion of the distance required to adjust to a bathymetric fluctuation is presented by (Horritt, 2002) and in Appendix A1. Figure 9 also shows that the fluctuations, $\epsilon_{S_{f}}$ and *ϵ*_*H*_, have a complicated nonlinear relationship which depends on both discharge and riffle‐pool wavelength. Figures (7) and (8) show solutions for *H*, *S*_*f*_, *U* ^2^/2*g*, *F*_*r*_ for a wavelength ranging from fast small riffles to slower and longer variations. They also present (dashed lines, lower left) the kinetic energy head that would be obtained if *U* is estimated using the slope of the water surface elevation as an approximation for *S*_*f*_ (diffusion approximation). For the 4 km smoother, longer wavelength variations, and away from very large discharges, the diffusion approximation gives good estimates for the kinetic energy head. However, while the estimates are not unreasonable for lower discharges when the riffle‐pool wavelength is 1 km, significant high‐frequency differences arise in the diffusion wave estimate of the kinetic energy  head.

To assess the hydraulic visibility of these systems (in the absence of measurement noise), we define hydraulic visibility as the ability to invert the Saint Venant equation exactly by iterating the solutions of the Saint Venant equation given the observables (i.e., surface slope and elevation in this case), as described in section 3. In the middle right panels of Figures 7 and 8 we plot using solid lines the −*∂*_*x*_ *E*_*k*_=*S*_*f*_−*S*_*h*_ diffusive term obtained from the numerical solution of the equation, which, as expected, is much greater for the shorter wavelengths. On the same panel, we plot as dashed lines the residual *S*_*f*_−*S*_*h*_+*∂*_*x*_ *E*_*kM*_, where *E*_*kM*_ is the kinetic energy head estimated using the Manning equation under the diffusive approximation; that is, this is a plot of the hydraulic visibility of this riffle‐pool sequence in the absence of noise. We see that the longer wavelength system is fully observable (i.e., the curves agree with each other), as are most of the middle to lower discharge conditions for the faster variations and conclude that, in the absence of noise and for many situations, the scheme proposed in section 3 will result in the hydraulic visibility of the riffle and pool sequence examined here, and hydraulic visibility is determined by instrument elevation noise. In the middle right panels, we also examine whether reach averaging will result in a system that is fully observable at scales longer than the riffle‐pool wavelength. The panels present the result of averaging −*∂*_*x*_ *E*_*k*_ over one wavelength: The residual represents the validity of the diffusion wave approximation after reach averaging. As expected, the variability of the advective term is concentrated at small wavelengths and mostly averages out (at better than ∼1 cm/km) for most of the cases studied: This shows the promise of reach averaging for hydraulic visibility at larger scales.

We have also used this sample system to validate that, when the within‐reach parameters are known, equation 19 predicts the difference between the true discharge and the discharge obtained by using the reach‐averaged parameters without modifying the friction coefficient: We find that the analytic result is accurate to better than four significant digits in predicting *κ*_*T*_. We examine next the efficacy of the two approximations, equations 31 and 30, in predicting the change in the friction coefficient required to estimate the discharge using the average *H* and *S*_*f*_; the results are presented in Table 6. This table shows that both estimates do a reasonable job in predicting *κ*_*T*_, especially when the parameter variability is <1. Even when the parameter variability is large, the errors are only in the 20% to 30% range. The lognormal distribution estimates are not unreasonable even though the parameters are far from lognormal, and it tends to overestimate *κ*_*T*_, while the other approximation underestimates *κ*_*T*_. In general, the variability of *S*_*f*_ dominates, but *H* variability cannot be ignored. In the next section, we examine more realistic river conditions.

**Table 6:** Fluctuation and *κ* Friction Increase Factor for the Riffle and Pool Toy Model, as a Function of Riffle/Pool Wavelength, *L*, and Discharge, *Q*

				*κ*_*T*_	*κ*_*TA*_	*κ*_*TA*_	*κ*_*TL*_	*κ*_*TL*_
*L* (km)	*Q* (m^3^/s)	$\bar{\epsilon_{S_{f}}^{2}}$	$\bar{\epsilon_{H}^{2}}$	True	Approx.	% Error	Lognormal	% Error
0.5	25	0.45	0.03	0.13	0.14	7.33	0.15	14.94
	50	0.24	0.02	0.07	0.07	5.10	0.08	9.07
	100	0.12	0.01	0.04	0.04	2.86	0.04	4.76
	200	0.06	0.00	0.02	0.02	1.27	0.02	2.18
	400	0.03	0.00	0.01	0.01	0.10	0.01	0.54
1.0	25	0.91	0.05	0.26	0.27	3.86	0.31	19.16
	50	0.65	0.04	0.18	0.19	7.84	0.21	18.85
	100	0.39	0.02	0.11	0.12	7.30	0.12	13.80
	200	0.20	0.01	0.06	0.06	4.74	0.06	8.11
	400	0.10	0.01	0.03	0.03	3.13	0.03	4.79
4.0	25	1.46	0.09	0.54	0.44	‐19.06	0.55	1.55
	50	1.37	0.08	0.45	0.41	‐10.61	0.50	10.24
	100	1.23	0.06	0.36	0.36	‐0.93	0.43	19.27
	200	1.03	0.05	0.28	0.30	8.66	0.35	26.67
	400	0.79	0.04	0.20	0.23	15.88	0.26	30.21
10.0	25	1.40	0.10	0.61	0.44	‐28.44	0.55	‐10.20
	50	1.34	0.09	0.53	0.41	‐22.98	0.51	‐4.73
	100	1.25	0.08	0.45	0.38	‐16.54	0.46	1.51
	200	1.14	0.07	0.38	0.34	‐9.27	0.41	8.22
	400	1.02	0.06	0.31	0.30	‐1.57	0.35	14.84

*Note*. The third and fourth columns report the normalized hydraulic parameter variability results from the model. The fifth column reports the observed value for *κ*_*T*_ from the model. The predictions using the small (equation (31)) or lognormal (equation (30)) approximations are given in the sixth and eighth columns, and their percentage deviation from the true value is reported alongside.

### 5.3 Results Using Calibrated River Models

**Figure 10:**
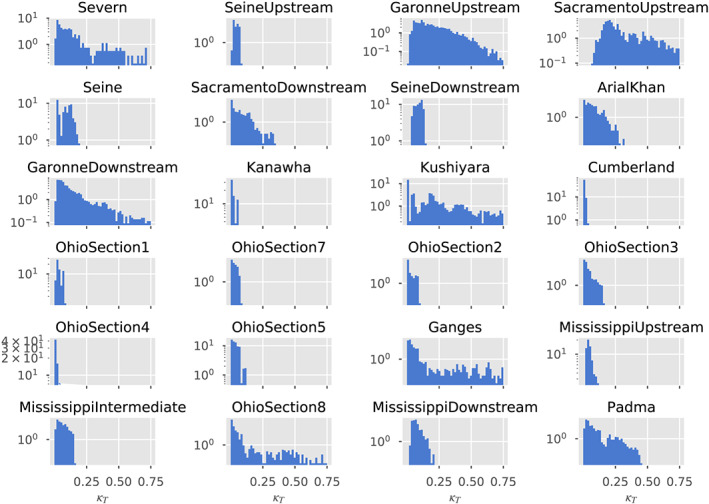
Log histograms of the friction increase factor, *κ*, for the rivers in this study. While some rivers have a narrow range of variability, others have significant tails in the distribution.

**Figure 11:**
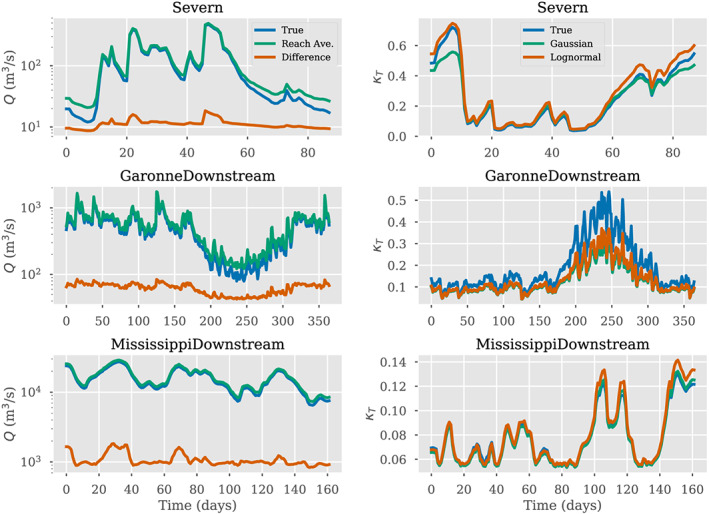
(Left column) Sample hydrographs for one reach in three high variability representative rivers. The true reach‐averaged discharge is given in blue, the discharge estimated using the average hydraulic parameters over the reach is given in green, and the difference between the two curves is given in orange. (Right column) True value of *κ*_*T*_=*Q*_*A*_/*Q*_T_−1 (blue) and values estimated using equation (31) (green) or equation (30) (orange).

**Figure 12:**
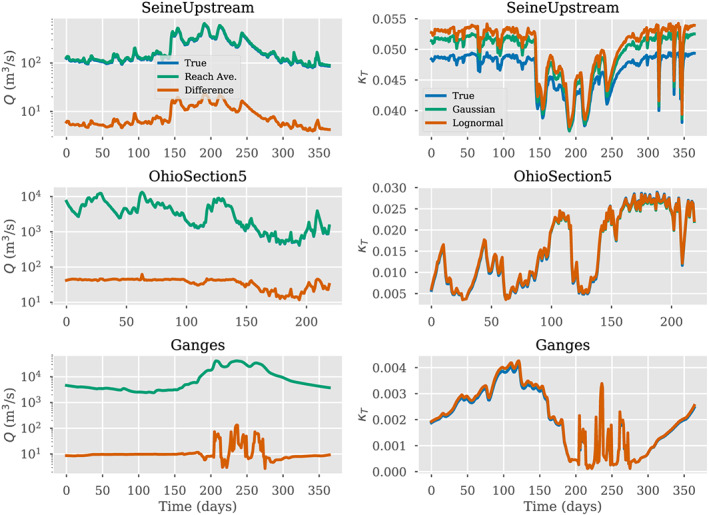
Same as Figure 11 but for representative low‐variability rivers.

We reach‐averaged the models described in section (2) and computed *κ*_*T*_=*Q*_*A*_/*Q*_*T*_−1, where *Q*_*T*_ is the true discharge and *Q*_*A*_ is the discharge obtained by using the average hydraulic parameters in the Manning equation, without adjusting the Manning coefficient. Histograms of the distribution of *κ*_*T*_ for all of the rivers and all times are presented in Figure 10, and they are organized by discharge (left to right, top to bottom). The results show no significant trend with median discharge: Some rivers have a very narrow range of values for *κ*_*T*_, usually below 0.1, while some others exhibit significant tails, with extreme values of *κ*_*T*_ greater than 0.5. To gain greater insight about the source of variability, we show in Figures 11 and 12 hydrographs for reaches with small and large variability, respectively. We see that, for most of the time, *κ*_*T*_ is on the order of 0.1 for the rivers with large variability but can increase substantially in low flow situations, and it is in these cases where we find the greatest disagreement between the statistically estimated *κ*_*T*_ and the true value. At least in these examples, there is little difference between the lognormal error estimate and the one for small fluctuations. For some of the smoother rivers, *κ*_*T*_ can be significantly below 0.05, and either estimate predicts it quite  well.

**Figure 13:**
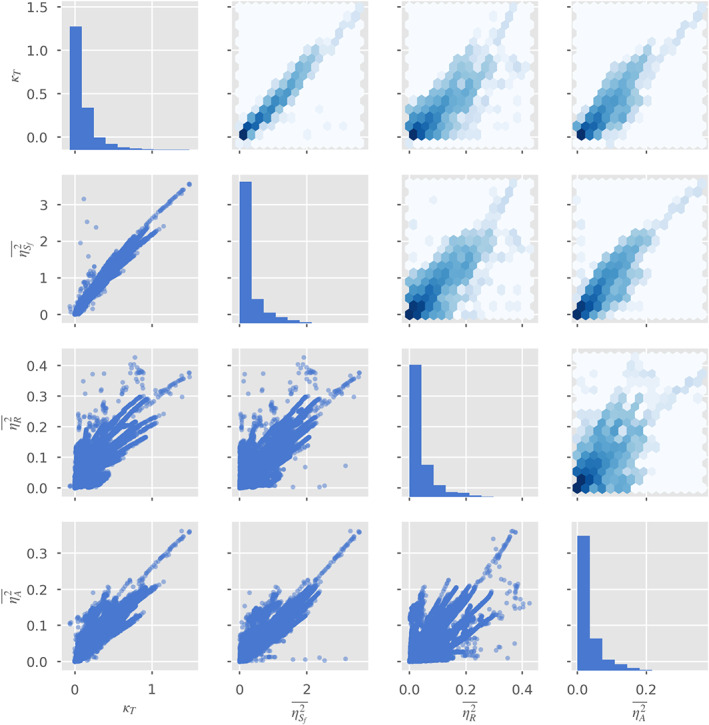
Pair plots of the distribution of *κ*_*T*_ and the fluctuations of the log variables, *η*_*Sf*_, *η*_*R*_, and *η*_*A*_. Below the diagonal, scatterplots of the variable pairs are shown, while above the diagonal shows hex‐bin plots in logarithmic scale that provide a better visualization of the density and location of outliers. The diagonal has the histograms of the fluctuations, which show strong tails.

**Figure 14:**
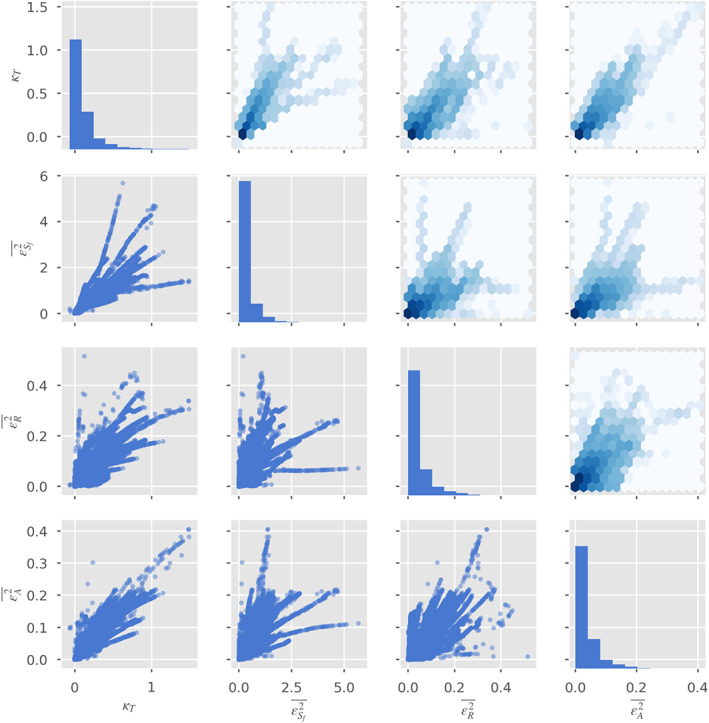
Same as Figure 13 but for the linear fluctuations, *ϵ*_*Sf*_, *ϵ*_*R*_, and *ϵ*_*A*_. In contrast to Figure 13, there is much greater scatter in the relationship, consistent with observations in Figure 9.

In Figure 13 we examine the relationship among the log parameter fluctuations and *κ*_*T*_. The dominant factor in determining *κ*_*T*_ is the variability of the log of *S*_*f*_, and there is a near‐linear dependence between the two. The contribution from the other parameters is almost an order of magnitude smaller, although they also exhibit a strong correlation with *κ*_*T*_. The correlation between parameters is strongest with pairs involving *S*_*f*_, while the correlation between *A* and *R* is significantly weaker. Figure 14 presents a similar plot for the correlation with the linear parameters. As expected, the spread in the relation shows significantly greater spread as the magnitude of the variability increases. The strongest dependence of *κ*_*T*_ is still on *S*_*f*_, but the histograms show significant departures from linearity at times. The covariance between the linear parameters shows significant spread and drops in correlation. This is similar to what was observed in Figure 9.

**Figure 15:**
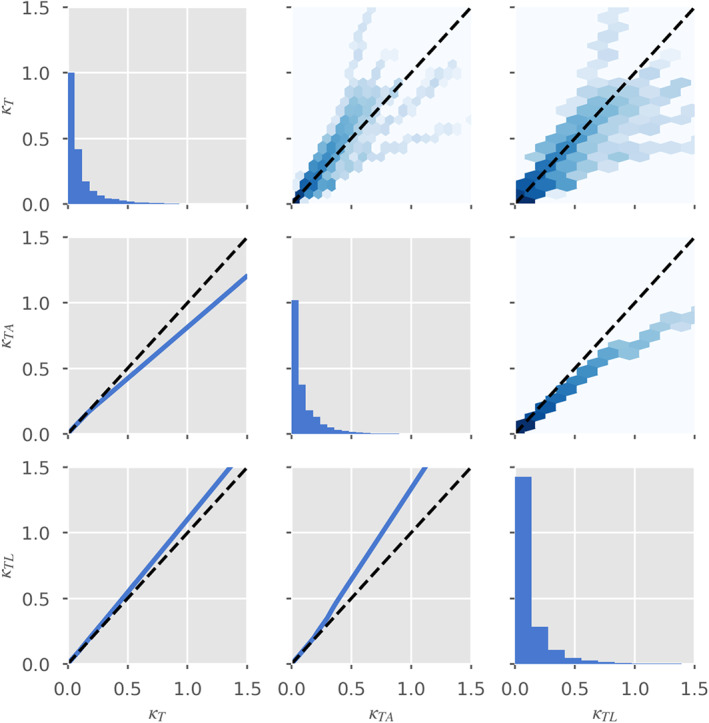
Pair plots of the relationship between the true friction factor, *κ*_*T*_, and values estimated using *κ*_*TA*_ (equation (31)) or *κ*_*TL*_ (equation (30)). Below the diagonal are shown LOESS (Cleveland & Devlin, 1988) fits through the data, while the hex‐bin plot of the log‐distribution are shown above the diagonal.

In Figure 15 we assess how well the two statistical estimates approximate the observed increase in the friction coefficient. This figure shows that for small values of *κ*_*T*_, equations 31 and 30 both do quite well in the mean, but as the variability increases the lognormal estimate from equation (30) is a much better predictor of *κ*_*T*_. In general, the lognormal estimate will overpredict a little, while the other estimate will underpredict.

**Figure 16:**
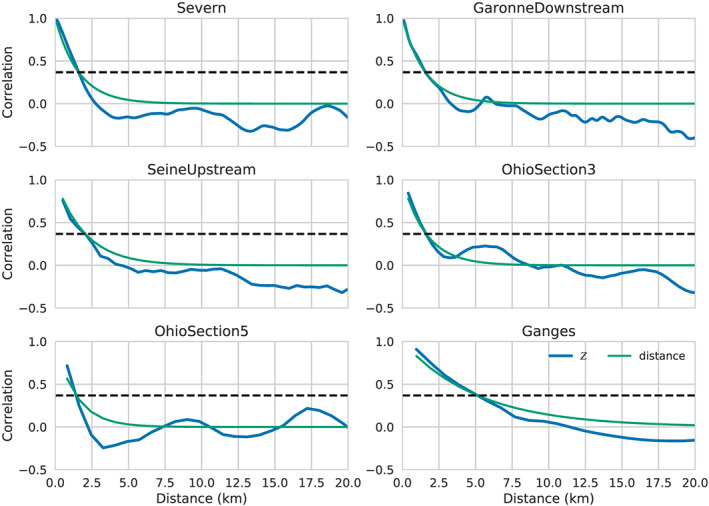
(blue) Estimated bathymetry, *Z*, correlation function for selected rivers as a function of distance. (green) Exponential correlation fit adjusted to match 1/*e* crossing, which defines *L*_*Z*_.

While *κ*_*T*_ can be calculated exactly if the subreach variability is known, estimates of subreach variability are currently only feasible for the width, where optical sensors provide high‐resolution imagery (Allen & Pavelsky, 2018). Unfortunately, as shown in Figure 13, the slope variability dominates by almost an order of magnitude contributions to *κ*_*T*_, and no global data set of high‐resolution river slopes exists at this time. In the absence of subreach variability data, it is desirable to be able to relate the variability to parameters that are observable at reach scale. As a first step, in Appendix A1 we elaborate on an approach proposed by Horritt (2002) and Li et al. (1992) to examine two cases wherein the parameter variabilities can be directly related to the reach‐averaged variables in the steady gradually varying approximation: When bathymetry fluctuations dominate, or, conversely, are dominated by, width fluctuations, we show there that the spectra of fluctuations of depth, surface elevation, and slope can all be expressed using transfer functions applied to the spectra of bed elevation or width fluctuations. Horritt (2002) and Li et al. (1992) propose that the correlation function for the bed fluctuations can be approximated by an exponential; that is, $C_{Z|W}(x)=\exp\left[-|x|/L_{Z|W}\right]$, where *L*_*Z*_ and *L*_*W*_ are correlation lengths characterizing the bed elevation or width fluctuations (i.e., after removing long‐wavelength secular terms), respectively. We show in Figure 16 that this a good approximation for the bathymetry in the models used in this study and present the estimated *L*_*Z*_ for each river in Table 3. In Figure 5, we see that the width, and other parameter, fluctuations have similar scales and shapes, showing their potential dependence on the bed bathymetry.

When the bathymetry fluctuations dominate, we obtain the following closed form solution for the normalized parameter variabilities:

(39) $$\bar{\epsilon_{R}^{2}}={\left(\frac{\sigma_{Z}}{\bar{R}}\right)}^{2}\frac{1}{1+\rho_{Z}}\frac{1}{{\left(1-\bar{F_{r}^{2}}\right)}^{2}}$$

(40) $$\bar{\epsilon_{S_{f}}^{2}}=a^{2}\bar{\epsilon_{R}^{2}}$$

where $\sigma_{Z}^{2}$ is the variance of the bed elevation fluctuations, *a*=10/3 assuming a friction slope given by the Manning relation. We have introduced *ρ*_*Z*_=*L*_*Z*_/*L*_*fH*_, where *L*_*fH*_>0, given by equation (A4), is the length required for the downstream decay of river bed fluctuations to decrease by 1/*e*, which we show is approximately equivalent to the downstream length required for the bed elevation to decrease by one third of the mean depth. Table 3 shows the median and median absolute deviation for *L*_*fH*_, and we see that, aside from a few high slope rivers (e.g., the Severn), *ρ*_*Z*_≪1 and it can be neglected in equation (39), leading to the result that the normalized depth variability is approximately equal to the ratio of the bed elevation fluctuation variance scaled by the depth squared, with a small dependence on the Froude number. As an additional surprising result, equation (40) shows that, in the bathymetry dominated limit, the normalized slope variability is directly proportional to the normalized depth variability, with a proportionality constant that is *a* ^2^≈11, or an order of magnitude higher.

In the opposite limit, when width variations dominate bathymetry variations, the normalized parameter variabilities are given by:

(41) $$\bar{\epsilon_{R}^{2}}=\frac{4}{a^{2}}{\left(\frac{\sigma_{W}}{\bar{W}}\right)}^{2}\frac{\rho_{W}}{1+\rho_{W}}$$

(42) $$\bar{\epsilon_{S_{f}}^{2}}=a^{2}\rho_{W}\bar{\epsilon_{R}^{2}}$$

where $\sigma_{W}^{2}$ is the width variance and *ρ*_*W*_=*L*_*W*_/*L*_*fH*_≪1. Comparing equation 42 with 40, we see that, when width variations dominate, there is a much weaker relationship between the normalized slope and depth variations, so the ratio between the two is a good indicator of whether the variabilities are dominated by variations of bathymetry or width. The normalized depth and slope variabilities will both be proportional to ${\bar{W}}^{-2}$, so should have a greater impact on *κ*_*T*_ for narrower rivers, given constant width variations.

**Figure 17:**
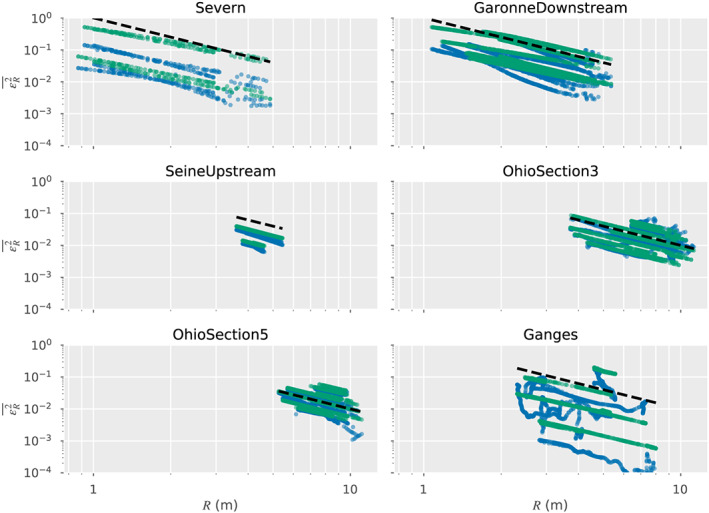
Dependence of the hydraulic radius normalized variability, $\bar{\epsilon_{R}^{2}}$, as a function of hydraulic radius, *R*, for selected rivers. (green) Theoretical predictions. (blue) Model observations. (dashed line) *R* ^−2^ line, showing the slope predicted by theory when *ρ*≪1. Different lines of the same color in each figure represent different reaches as a function of time. The *R* ^−2^ behavior is followed in most cases but deviates for high slope rivers (e.g., Severn) when ρ∼O(1), when it is shallower, as predicted by simple theory.

**Figure 18:**
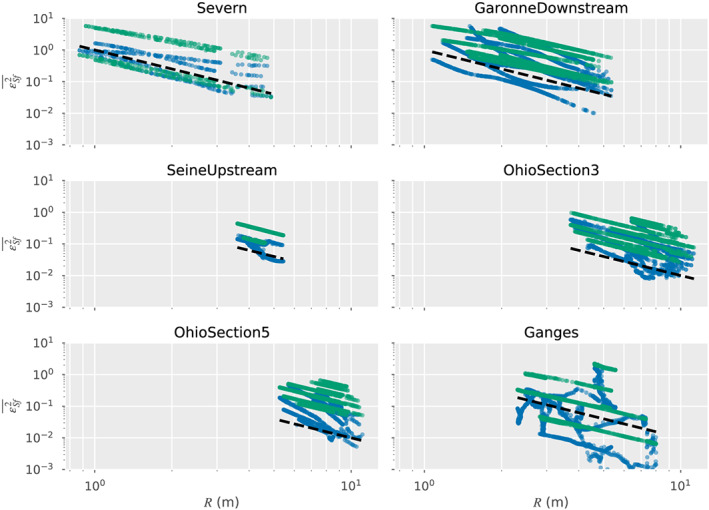
Dependence of the friction slope normalized variability, $\bar{\epsilon_{Sf}^{2}}$, as a function of hydraulic radius, *R*, for selected rivers. (green) Theoretical predictions. (blue) Model observations. (dashed line) *R* ^−2^ line, showing the slope predicted by theory when *ρ*≪1. Note that the slope variability is about an order of magnitude greater than the hydraulic radius variability and matches qualitatively the shape. The differences in the shape of the two curves should be due to the width variability, unaccounted for in the theory.

As a first indication of which limit applies, we examine the distribution of normalized variabilities in Figure 14 and see that in general $\bar{\epsilon_{S_{f}}^{2}}$ is approximately an order of magnitude larger than $\bar{\epsilon_{R}^{2}}$, although a detailed comparison (not shown) shows that it generally falls below the *a* ^2^≈11 factor predicted by the bathymetry dominated limit, indicating that, for certain rivers, width variations need to be taken into consideration. For a more quantitative assessment, we compare the predictions assuming bathymetry dominated fluctuations to the observations for representative rivers in Figures 17 and 18. These figures show that even when width fluctuations are present, the normalized variabilities decay mostly as ${\bar{R}}^{-2}$, with some deviations for the Severn, when *ρ*_*Z*_ cannot be neglected. The figures also show the similarity in behavior between depth and slope fluctuations, as well as the scale difference which is close to an order of magnitude. The actual predicted values agree well for some rivers, while theory can overpredict the observed value for others (e.g., Ganges), presumably because width variations are an important part of the variability for wide, low‐slope rivers.

**Figure 19:**
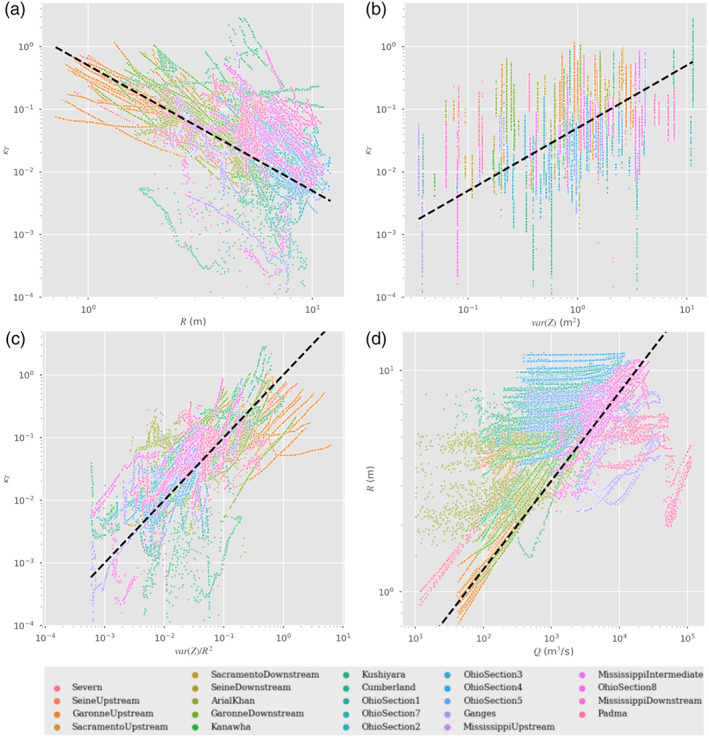
(a) *κ*_*T*_ as a function of hydraulic radius, *R*, color coded by river segment, ordered by discharge. The dashed line is proportional to *R* ^−2^, as predicted by theory for *ρ*≪1. Although there is variability, due to different bathymetry friction and width variations, most traces exhibit behavior well approximated by *R* ^−2^. (b) *κ*_*T*_ as a function of bathymetry variance, $\sigma_{Z}^{2}$, within the reach. Different reaches align horizontally, since the bathymetry is time independent. The dashed line is proportional to $\sigma_{Z}^{2}$, as predicted by theory. (c) *κ*_*T*_ as a function of $\sigma_{Z}^{2}/R^{-2}$, which should be linear when *ρ*≪1. (d) *R*‐*Q* hydraulic relation for all the rivers in the study, overlaid with a *Q* ^0.4^ line. There is significant variability in the exponent of the hydraulic relationship for different rivers, which leads to a dependence on *Q* that is not simple. When the exponent is 1/2, *κ*_*T*_∼*Q* ^−1^.

Given these bathymetry dominated results, we expect that *κ*_*T*_ might scale as $\sigma_{Z}^{2}/{\bar{R}}^{2}$. In Figure 19, we examine the behavior of *κ*_*T*_ as a function of these variables and conclude that *κ*_*T*_ indeed has the expected dependence on *σ*_*Z*_ and $\bar{R}$, but the overall magnitude of the variability still has some dispersion around the simple model, presumably due to the neglected neglected width variability or to assuming that *ρ*_*Z*_≪1. When the variability is plotted as a function of discharge (not shown), we do not observe the same simple relation observed with depth. This can be understood as being due to the variability in the at‐a‐station hydraulic relation between depth and discharge (Leopold & Maddock, 1953; Singh, 2003). While for many of the rivers in our sample, the *f* exponent in the hydraulic relation *R*∼*Q* ^*f*^ seems to be around values of 0.4–0.5, typical of many rivers (Singh, 2003), there is a significant number of rivers in our sample (e.g., the Ohio) where a much smaller value is observed. When *f*∼0.5, *κ*_*T*_∼*Q* ^−1^, but this behavior is not always as evident as the dependence on the depth and bathymetry variance.

**Figure 20:**
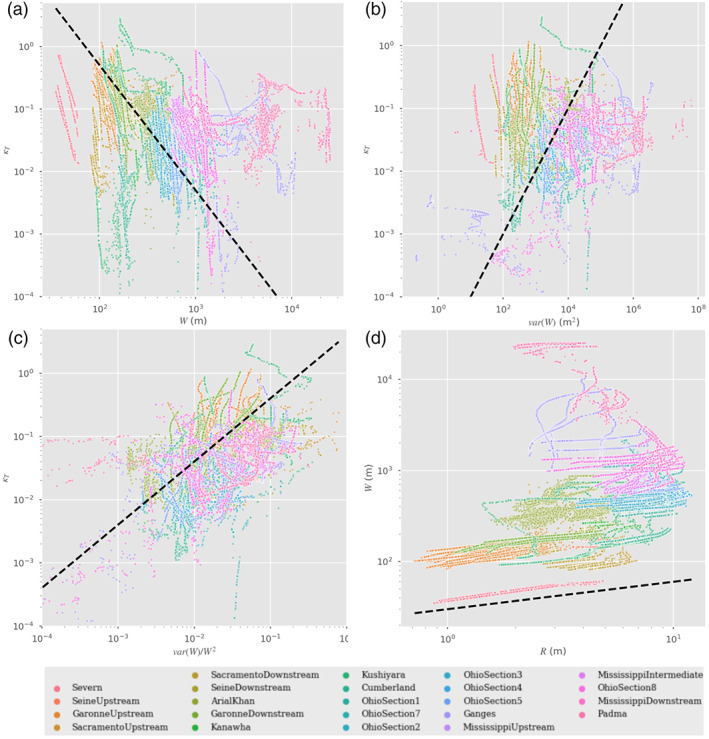
(a) *κ*_*T*_ as a function of width, *W*, color coded by river segment, ordered by discharge. The dashed line is proportional to *W* ^−2^, as predicted by theory for *ρ*≪1. (b) *κ*_*T*_ as a function of width variance, $\sigma_{W}^{2}$, within the reach. The dashed line is proportional to $\sigma_{W}^{2}$, as predicted by theory. (c) *κ*_*T*_ as a function of $4\sigma_{W}^{2}/W^{-2}$, which should be linear when *ρ*≪1. (d) *W*−*R* hydraulic relation for all the rivers in the study, overlaid with a *R* ^0.3^ line.

In Figure 20 we present the dependence of *κ*_*T*_ on width and width variability. Although there is significantly greater dispersion relative to the results in Figure 19, Figure 20c shows that, in general, $\kappa_{T}∼{\left(\sigma_{W}/\bar{W}\right)}^{2}$, as predicted by the width fluctuation limit. However, in general, the fluctuations between width and depth are strongly correlated due to the interdependence of the at‐a‐station discharge relations (Leopold & Maddock, 1953; Singh, 2003). Figure 20d shows the relationship between depth and width for our data set, which indicates a clear power law relationship, which may vary between rivers but is generally well approximated by $\bar{W}∼{\bar{R}}^{0.3}$. The presence of this relation shows that the detailed correlation between depth and width (which depends on river and season) must be taken into consideration when predicting the behavior of *κ*_*T*_. Nevertheless, given the close agreement between predicted and observed normalized variabilities, we conclude that the bathymetry dominated fluctuations are a good guide to the general behavior and that width fluctuations may be implicitly accounted for in the bathymetry fluctuations or, if independent, may play a secondary role in the behavior of *κ*_*T*_.

The net effect of reach averaging is that the product $n\left(1+\kappa_{T}\right)$, which has the effect of acting as an effective friction coefficient in the reach‐averaged Saint‐Venant equations, will have a form

(43) $$n\left(1+\kappa_{T}\right)=n\left(1+\frac{\nu }{{\bar{R}}^{2}}\right)$$

where *ν* is a parameter that depends on the bed and width variabilities. This means that calibrating for a Manning coefficient while neglecting the subreach variability will result on a depth‐varying Manning coefficient. For small fluctuations, we expect that this dependence would not be noticeable for large depths but might become apparent as the river depth decreases. The high‐resolution hydrodynamic models in this study nearly all used a constant friction coefficient, but our results show that calibrating a discharge model using reach‐averaged data must take into account the fact that the effective friction coefficient varies with depth, which depends on discharge. This dependence is not due to changes in the rate of energy loss at the walls of the river but in kinetic energy loss within the unobserved reach by parameter variability that increases in low flow conditions. As we saw previously, this increase is mainly due to the increase in the friction slope variability during low flow, when the water surface elevation slope reflects the rough bottom bathymetry more closely (see Horritt, 2002, and Appendix A1).

We note that the discharge dependence of Manning's coefficient is comparable to changes obtained from calibrated models of the Tiber and Po Rivers in Italy (Domeneghetti et al., 2012; Moramarco & Singh, 2010). The reach length used by Moramarco and Singh (2010) was ∼4 km, while the ones used by Domeneghetti et al. (2012) were longer. Both of these studies report nearly constant asymptotic behavior for Manning's *n* for discharge value of 10^3^ m^3^/*s* in the Po River (Domeneghetti et al., 2012) or flow depths greater than ∼1–1.5 m in the Tiber. For smaller discharges or flow depths, they noted a sharp increase in the calibrated Manning coefficient. Given the degree of dispersion of the points in Figure 19, one must take the exact numbers with care, as we expect them to vary from river to river, depending on the variability of bathymetry and width. Nevertheless, reach averaging may be partly responsible for the observed variability in the calibrated friction coefficient. We conclude that care must be exercised in the inversion of SWOT data to retrieve discharge, friction and bathymetry to take this effect into account. Using an earlier version of this work, we showed in Durand et al. (2016) that the variability of the reach‐averaged Manning coefficient with discharge impacted the accuracy of the Metropolis‐Manning (MetroMan) discharge estimation algorithm and suggested that future versions should take this into consideration.

## 6 Discussion and Conclusions

We have presented a unified framework for using noisy, spatially averaged remote sensing data within a set of dynamic equations that are nearly identical to the Saint Venant equations but use as dynamical variables the reach‐averaged variables, rather than the unobserved point variables. This process allows for the consistent use of different resolution instruments and presents dynamic equations that can be used for upscaling applications. Our main finding regarding the dynamic equations is that within‐reach energy loss not captured by the spatially smoothed parameters must be accounted for by a single additional scaling factor, proportional to the weighted sum of the within‐reach parameter variances. Since this factor is multiplicative and represents kinetic energy loss, we suggest that it be incorporated into an effective friction coefficient which is generally greater than the friction coefficient measured at a cross section. An additional factor, similar to the Boussinesq term but accounting for kinetic energy variations within a reach, also appeared to modify the diffusive term of the Saint Venant equation. A connection between energy conservation at two cross sections and the energy loss due to friction and within‐reach variability was also established. This relationship hinted that reach‐averaged discharge was in fact calibrated when using energy conservation at two cross sections.

We also addressed the hydraulic visibility (Garambois et al., 2017) of the Saint Venant equation, given typical instrument noise. We found that, in the absence of noise, the data to be collected by the SWOT mission would be sufficient to observe both diffusion and advective wave terms in the Saint‐Venant equation. However, in the presence of noise, the hydraulic resolution capability is determined by random errors in measuring the surface slope. For high‐slope rivers, kilometer‐scale variations could be observed, but lower slope rivers must often be observed at scales greater than or equal to 10 km. By using the PSD of the hydraulic variables, we determined that, given expected achievable noise levels by SWOT, only the diffusion wave term of the Saint‐Venant equation is hydraulically observable, while the advective term lies outside the scale of observability. This conclusion agrees with Garambois and Monnier (2015), who found numerical instability when the advective term was kept in the Saint‐Venant equation.

We also examined the transition to a statistical description of the within‐reach energy dissipation and found that, for most rivers, statistical stability could be expected for 10 km reaches, although some reaches had longer correlation lengths. We showed that if we assumed that the hydraulic parameters are lognormal, then a good estimate of the increase in the friction coefficient could be obtained knowing only the variability of the parameters within the reach. This shows the importance of using high‐resolution instruments to parametrize reach variability below 10 km scales. A good step in this direction is the characterization of global river width by Allen and Pavelsky (2018). However, the strongest determinant in the increase of friction is the water surface slope, which, in the kinematic wave approximation, follows the bed slope. One approach to obtaining bed slopes is to use existing DEMs (Dai et al., 2018; Yamazaki et al., 2014). Another approach, which holds much promise, is to use the long‐term data from the SWOT mission to map the average water surface topography and slope. Although for any given pass the data will be noisy, temporally combining the data will yield a static map that will be useful in characterizing slopes at spatial resolutions smaller than 10 km.

As a final result, we showed that, even when the point friction coefficient is a constant, the reach‐averaged coefficient can show significant dependence on discharge, especially during low‐flow conditions. This is similar to what is observed when calibrating discharge models (Domeneghetti et al., 2012; Moramarco & Singh, 2010) but it is not clear whether these two observations are related. As pointed out by an anonymous reviewer, the Hicks and Mason (1991) “field data show that in many natural rivers, the value of Manning's *n* increases with smaller values of Froude number and Reynolds number. This indicates that flow resistance varies with the state of the flow. The more the state of flow is dominated by gravitational and viscous forces, the more flow resistance increases.” Our results show that, even if there is dependence on the discharge due to physical reasons, a significant additional dependence will appear due to reach averaging. This discharge dependence must be taken into account for optimal estimation of river discharge from SWOT data, as pointed out by Durand et al. (2016).

## Supporting information



Supporting Information S1

## Data Availability

The data used in this study are openly available from Frasson et al. (2019) under a Creative Commons Attribution License.

## Appendix A Variability and Reach‐Averaged Parameters

### A.1

We extend the approach used by Horritt (2002) and Li et al. (1992) to apply to uniform gradually varying flow in rectangular channels of varying width. We assume that depth, *H*, width, *W*, and bathymetry, *Z*, can be divided into unperturbed components, $\bar{H}$, $\overset{\leftarrow }{W}$, and $\bar{Z}$, which satisfy the uniform gradually varying flow equation (Chaudhry, 2008)

(A1) $$\frac{dH}{dx}=\frac{1}{1-F_{r}^{2}}\left[\frac{dZ}{dx}-S_{f}+\frac{F_{r}^{2}}{W}H\frac{dW}{dx}\right]$$

exactly for the unperturbed geometry and zero‐mean fluctuations, *H* *′*, *Z* *′*, and *W* *′*, due to small‐scale fluctuations of the bed elevation or width, respectively. We expand equation (A1) to first order in the fluctuating components and assume that the Froude number is small, so that terms proportional to higher powers than $\bar{F_{r}^{2}}$ can be ignored, including within‐reach Froude number variations. The resulting equation is

(A2) $$\frac{dH^{\prime }}{dx}+\frac{1}{1-\bar{F_{r}^{2}}}\frac{\partial S_{f}}{\partial H}H^{\prime }=\frac{1}{1-\bar{F_{r}^{2}}}\left[\frac{dZ^{\prime }}{dx}-\frac{\partial S_{f}}{\partial W}W^{\prime }\right]$$

where we have neglected the last term in equation A1, which can be shown to be a generally negligible contribution using dimensional analysis and typical width fluctuations. Assuming the Manning relation, $S_{f}={\left(nQ/H^{5/3}W\right)}^{2}$, this equation becomes a simple linear inhomogeneous differential equation

(A3) $$\frac{dH^{\prime }}{dx}-\frac{H^{\prime }}{L_{fH}}=\frac{1}{1-\bar{F_{r}^{2}}}\frac{dZ^{\prime }}{dx}+\frac{W^{\prime }}{L_{fW}}$$

(A4) $$L_{fW}=\frac{\left(1-\bar{F_{r}^{2}}\right)\bar{W}}{2S_{f}}=\frac{a}{2}\frac{\bar{W}}{\bar{H}}L_{fH}\gg L_{fH}$$

where *a*=10/3. The length scale, *L*_*fH*_>0, is approximately given by three times the downstream distance required for the mean riverbed to drop by its mean depth, giving rise to a potential energy loss of $∼g\bar{H}/3$. The solution of the uniform differential equation also shows that *L*_*fH*_ is the length required for a perturbation to attenuate by 1/*e*. Although explicit closed‐form solutions exist, given *Z* *′* and *W* *′*, we are more interested in treating the fluctuations statistically and follow Horritt (2002) and Li et al. (1992) and solve the equation by taking its Fourier transform, and solving for $\tilde{H}(k)$, the Fourier coefficients of *H* *′*(*x*), in terms of Fourier coefficients of the riverbed, $\tilde{Z}(k)$, and width $\tilde{W}(k)$, fluctuations:

(A5) $$\tilde{H}(k)=\frac{-1}{ik+L_{fH}^{-1}}\left[\frac{ik\tilde{Z}(k)}{1-\bar{F_{r}^{2}}}+\frac{\tilde{W}(k)}{L_{fW}}\right]$$

Since the free surface is given by *h*=*H*+*Z*, and the free surface slope by *S*=*dh*/*dx*, one can solve for their Fourier coefficients

(A6) $$\tilde{h}(k)\approx \frac{L_{fH}^{-1}\tilde{Z}(k)}{ik+L_{fH}^{-1}}+\frac{\tilde{W}(k)}{L_{fW}\left(ik+L_{fH}^{-1}\right)}$$

(A7) $$\tilde{S}(k)=-ik\tilde{h}(k)$$

where we have assumed that *H* *′* and $F_{r}^{2}$ where of the same order to neglect a higher order  term.

We assume that the bed and width fluctuations can be treated as a homogeneous, stationary process, such that the bed and width fluctuation spectra, *F*_*Z*_(*k*) and *F*_*W*_(*k*), respectively, are given by

(A8) $$F_{Z}(k)=\left\langle \tilde{Z}(k){\tilde{Z}}^{*}(k)\right\rangle =\sigma_{Z}^{2}\int dx\;C_{Z}(x)e^{ikx}$$

(A9) $$F_{W}(k)=\left\langle \tilde{W}(k){\tilde{W}}^{*}(k)\right\rangle =\sigma_{W}^{2}\int dx\;C_{W}(x)e^{ikx}$$

where angular brackets denote the expectation value; *C*_*Z*_(*x*) and *C*_*W*_(*x*) are the bed and width correlation functions, obtained after removing the unperturbed long‐wavelength bathymetry and width, and $\sigma_{Z}^{2}$ and $\sigma_{W}^{2}$ are the variances of the bed and width fluctuations, respectively. A specific form of the bed and width fluctuation spectra must be assumed to get explicit results for the depth and slope spectra. We show in Figures 16 and 5 that an exponential correlation function, $C_{Z|W}(x)=\exp\left[-\left|x\right|/L_{Z|W}\right]$, where *L*_*Z*_ and *L*_*W*_ are the bathymetry and width correlation lengths, respectively, is a good approximation for both the bed and width fluctuations. Replacing in equations (A8) and (A9), the bed and width spectra, *F*_*Z*_ and *F*_*W*_, are given by $F_{Z|W}=2L_{Z|W}\sigma_{Z|W}^{2}/\left(1+{\left(kL_{Z|W}\right)}^{2}\right)$.

The spectra for depth, elevation, and slope fluctuations are computed by taking the complex magnitude of equations A5–A7 and taking the expectation value of the results. In addition to terms proportional to *F*_*Z*_ and *F*_*W*_, their spectra will contain terms proportional to the cross‐spectra between bed bathymetry and width fluctuations, $\left\langle \tilde{Z}(k){\tilde{W}}^{*}(k)\right\rangle$, and its complex conjugate. These cross‐spectral terms are the Fourier transforms of the cross‐correlation function between bed bathymetry and width fluctuations, and we expect these to vary with river type; For example, in riffle and pool reaches, we expect the river width to increase in the pools with an associated change in river bottom fluctuations. Examining our data set, we find that, while width and bed friction can show significant correlations, this correlation is not universal and may even change substantially with river stage for the same river. To simplify the problem, we examine two limiting cases, that is, when either bed or width fluctuations dominate and the contribution from the nondominant term can be neglected.

When the width fluctuations can be neglected, we obtain the variance of depth and slope by using the Wiener‐Khinchin theorem to integrate their spectra over all frequencies

$$\begin{array}{ccc} \sigma_{H}^{2} & =\int_{-\infty }^{-\infty }dk\;\frac{2L_{Z}\sigma_{Z}^{2}}{\left(1+{\left(kL_{Z}\right)}^{2}\right)}\frac{1}{{\left(1-\bar{F_{r}^{2}}\right)}^{2}}\frac{{\left(kL_{fH}\right)}^{2}}{\left(1+{\left(kL_{fH}\right)}^{2}\right)} & \\ \sigma_{S}^{2} & =\int_{-\infty }^{-\infty }dk\;\frac{2L_{Z}\sigma_{Z}^{2}}{\left(1+{\left(kL_{Z}\right)}^{2}\right)}\frac{k^{2}}{\left(1+{\left(kL_{fH}\right)}^{2}\right)} \end{array}$$

These integrals can be simplified to obtain the following expressions for the normalized depth and slope variabilities, $\bar{\epsilon_{H}^{2}}$ and $\bar{\epsilon_{S}^{2}}$, respectively:

$$\begin{array}{ccc} \bar{\epsilon_{H}^{2}} & =\frac{\sigma_{H}^{2}}{{\bar{H}}^{2}}=\frac{2\sigma_{Z}^{2}}{{\bar{H}}^{2}{\left(1-\bar{F_{r}^{2}}\right)}^{2}}I_{2} & \\ \bar{\epsilon_{S}^{2}} & =\frac{\sigma_{S}^{2}}{{\bar{S}}^{2}}=\frac{2\sigma_{Z}^{2}}{{\bar{S}}^{2}L_{Z}^{2}}\rho_{Z}^{2}I_{2}\\ I_{2} & =\frac{1}{2\pi }\int_{-\infty }^{\infty }\frac{dk\;k^{2}}{\left(k+i\rho \right)\left(k-i\rho \right)(k+i)\left(k-i\right)} \end{array}$$

where *ρ*_*Z*_=*L*_*Z*_/*L*_*fH*_. The integral has two simple poles in the upper‐half complex plane and is easily evaluated using contour integration

(A10) $$I_{2}=\frac{1}{2\left(1+\rho_{Z}\right)}$$

Replacing this in the previous equations, and making the approximation valid for wide rivers in the diffusive wave approximation, $\bar{H}\approx \bar{R}$ and $\bar{S}\approx \bar{S_{f}}$,

$$\begin{array}{ccc} \bar{\epsilon_{R}^{2}} & ={\left(\frac{\sigma_{Z}}{\bar{R}}\right)}^{2}\frac{1}{1+\rho_{Z}}\frac{1}{{\left(1-\bar{F_{r}^{2}}\right)}^{2}} & \\ \bar{\epsilon_{S_{f}}^{2}} & =a^{2}\bar{\epsilon_{R}^{2}} \end{array}$$

which are the results used in the main  text.

In the opposite limit, when we can the width variations dominate the bathymetry variations, the surface elevation and depth slopes are identical, since *Z* *′*≈0, and, in analogy to the previous derivation, we find

$$\begin{array}{ccc} \sigma_{H}^{2} & ={\left(\frac{L_{fH}}{L_{fW}}\right)}^{2}\int_{-\infty }^{-\infty }dk\;\frac{2L_{W}\sigma_{W}^{2}}{\left(1+{\left(kL_{fW}\right)}^{2}\right)}\frac{1}{\left(1+{\left(kL_{fH}\right)}^{2}\right)} & \\ \sigma_{S}^{2} & ={\left(\frac{L_{fH}}{L_{fW}}\right)}^{2}\int_{-\infty }^{-\infty }dk\;\frac{2L_{W}\sigma_{W}^{2}}{\left(1+{\left(kL_{W}\right)}^{2}\right)}\frac{k^{2}}{\left(1+{\left(kL_{fH}\right)}^{2}\right)} \end{array}$$

which are easily integrated by contour integration, similarly to the *I*_2_ integral above:

$$\begin{array}{ccc} \sigma_{H}^{2} & =\sigma_{W}^{2}{\left(\frac{L_{fH}}{L_{fW}}\right)}^{2}\frac{\rho_{W}}{1+\rho_{W}} & \\ \sigma_{S}^{2} & =\frac{\sigma_{W}^{2}}{L_{W}^{2}}{\left(\frac{L_{fH}}{L_{fW}}\right)}^{2}\frac{\rho_{W}^{2}}{1+\rho_{W}}\\ \rho_{W} & =\frac{L_{W}}{L_{fH}} \end{array}$$

Using $L_{fH}/L_{fW}=2\bar{H}/(a\bar{W})$, the normalized depth and slope

$$\begin{array}{ccc} \bar{\epsilon_{R}^{2}} & =\frac{4}{a^{2}}{\left(\frac{\sigma_{W}}{\bar{W}}\right)}^{2}\frac{\rho_{W}}{1+\rho_{W}} & \\ \bar{\epsilon_{S_{f}}^{2}} & =4{\left(\frac{\sigma_{W}}{\bar{W}}\right)}^{2}\frac{1}{1+\rho_{W}}=a^{2}\rho_{W}\bar{\epsilon_{R}^{2}} \end{array}$$

## Appendix B Notation

### B.1

The notation used in this papers is presented in Tables B1 and B2.

**Table B1:** Notation Used in This Paper

Symbol	Definition
*A*	Wetted cross section
*α*_*i*_	*ith* hydraulic parameter exponent in equation (15)
*β*	Boussinesq momentum term, (Boussinesq, 1877)
*β*_*R*_	Reach fluctuation term, defined by equation (8)
*β*_*q*_	Lateral discharge fluctuation term, defined by equation (9)
*C*_*W*_	Width correlation function
*C*_*Z*_	Bathymetry correlation function
*E*_*k*_	Kinetic energy, *E*_*k*_=*U* ^2^/2*g*
*ϵ*_*i*_	Fractional within reach fluctuation for *p*_*i*_, defined by equation (25)
*F*(*x*)	Within‐reach discharge fluctuations, defined by equation (33)
*F*_*r*_	Froude number, $F_{r}=U/\sqrt{gH}$
*F*_*W*_	Width fluctuation spectrum, equation (A9)
*F*_*Z*_	Bathymetry fluctuation spectrum, equation (A8)
*g*	Acceleration of gravity, 9.8 m/s^2^
*H*	Depth of flow
*h*	Water surface elevation
*∂*_*x*_ *h*	Water surface downstream slope
*k*	Wavenumber in the *x* direction, *k*=2*π*/*λ*
*κ*_*i*_	Variability index for the *ith* hydraulic parameter, defined in equation (17)
*κ*_*T*_	Total variability index, defined by equation (19)
*L*_*Z*_	Width correlation length
*L*_*Z*_	Bathymetry correlation length
*L*_*fH*_	Length required for a depth perturbation to decay by 1/*e*, defined by equation (A4)
*λ*	Power spectral density wavelength
*n*	Manning friction coefficient
*N*_*p*_	Number of hydraulic parameters in the generic flow law
$\bar{p}$	Reach averaging of generic parameter *p*
*P*	Wetted perimeter
PSD	Power spectral density
*p*_*i*_	*ith* hydraulic parameter in equation (15)
*Q*	River discharge.
*q*	Lateral discharge.
*R*	Hydraulic radius, *R*=*A*/*P*.
*ρ*	Generic friction parameter.
*ρ*_*W*_	*L*_*W*_/*L*_*fH*_
$\tilde{\rho }$	Effective reach average friction parameter, equation (19).
*S*_0_	Magnitude of riverbed slope.
*S*_*h*_	Magnitude of water surface slope.
*S*_*f*_	Friction slope, defined in equation (1)
*σ*_*h*_	Water surface elevation measurement standard deviation.
*σ*_*h*_	River width measurement standard deviation.

**Table B2:** Notation Used in This Paper (Continued)

Symbol	Definition
*σ*_*H*_	Within‐reach depth standard deviation.
*σ*_*W*_	Within‐reach width standard deviation.
*U*	River velocity, *U*=*Q*/*A*.
*W*	River width.
*x*	River downstream distance.
*x*_*d*_	Downstream reach *x*‐boundary.
*x*_*u*_	Upstream reach *x*‐boundary.
*Z*_0_	Riverbed bathymetry.
*ζ*_*i*_	$\log\left(1+\epsilon_{i}\right)$.
