# Supplementary material for: Observing Rivers With Varying Spatial Scales
Source: Water Resour Res. 2020 Sep 14;56(9):e2019WR026476. doi: 10.1029/2019WR026476 (PMC7540328; doi:10.1029/2019WR026476)
Supplement: Supplementary file 1 — Supporting Information S1 [file WRCR-56-e2019WR026476-s001.pdf]

# Supporting Information for "Observing Rivers with Varying Spatial Scales"

Ernesto Rodríguez[1], Michael Durand[2], Renato Prata de Moraes Frasson[2]

[1]Jet Propulsion Laboratory, California Institute of Technology, Pasadena, CA, USA

[2]Byrd Polar and Climate Research Center, Ohio State University, Columbus, OH, USA

## Contents of this file

1. Text S1 to S5

## Introduction

In this supplement, we present mathematical details for the results presented in the paper.

## Text S1.

In this section, we give detailed derivations of the various forms of the Saint-Venant equations used in the text. The conventional writing of the momentum equation is in terms of the discharge (see, e.g., (Chaudhry, 2008), equation 12-15):

$$\frac{\partial Q}{\partial t} + \frac{\partial}{\partial x}(QU) + gA\frac{\partial H}{\partial x} = gA(S_0 - S_f) + u_x q \tag{1}$$

Corresponsing author: Ernesto Rodriguez, Jet Propulsion Laboratory, California Institute of Technology, 4800 Oak Grove Dr., Pasadena, CA 91109, USA. (ernesto.rodriguez@jpl.nasa.gov)

where $Q$ is the discharge; $A$ is the wetted cross section; $U$ is the flow velocity; $H$ is the flow depth; $S_0 = -\partial_x Z_0$ is the bed slope, and $Z_0$ is the bed elevation; $S_f$ is the friction slope; $q$ is the lateral inflow and $u_x$ is the downstream component of the lateral inflow velocity. In general, one can ignore the lateral inflow momentum (i.e., $u_x \approx 0$), even if the lateral inflow to the mass conservation equation is not negligible, and we will do so here.

-Using $\partial_x UQ = Q\partial_x U + U\partial_x Q$, dividing the previous equation by $gA$ and solving for $S_f$, one obtains

$$S_f = \underbrace{S_0}_{\text{I}} - \underbrace{\frac{\partial H}{\partial x}}_{\text{II}} - \underbrace{\beta \frac{\partial}{\partial x}\frac{U^2}{2g}}_{\text{III}} - \underbrace{\frac{1}{gA}\left(\frac{\partial Q}{\partial t} + U\frac{\partial Q}{\partial x}\right)}_{\text{IV}} \tag{2}$$

where we have introduced the Boussinesq momentum term, $\beta$, that parametrizes the *vertical* current variability contribution to the advected momentum (Boussinesq, 1877; Chow, 1959; Dingman, 2009).

Following Ponce and Simons (1977), and subsequent authors, one obtains the *kinematic wave* approximation if only the bed slope, term I, is retained. Retaining the bed slope and pressure (terms I and II) results in the *diffusive wave* approximation. Term III represents the loss of kinetic energy downstream, and adding it to terms I and II results in the *the steady dynamic wave*. Finally, keeping all the terms results in the *dynamic wave*.

Neither $Z_0$ nor $H$ can be measured easily using remote sensing, but the water surface elevation, $h = Z_0 + H$, can be measured using either radar interferometry (e.g., SWOT) or altimetry (lidar or radar). Combining terms I and II, the momentum equation can be written in a form more amenable to remote sensing observations:

$$S_f = \underbrace{-\frac{\partial h}{\partial x}}_{\text{Diffusive wave}} \underbrace{-\beta \frac{\partial}{\partial x} \frac{U^2}{2g}}_{\text{KE disipation}} \underbrace{-\frac{1}{gA}\left(\frac{\partial Q}{\partial t} + U\frac{\partial Q}{\partial x}\right)}_{\text{dynamic}} \tag{3}$$

The two terms in the final parenthesis can be rewritten as follows

$$\frac{1}{gA}\frac{\partial Q}{\partial t} = \frac{1}{gA}\frac{\partial AU}{\partial t} = \frac{1}{g}\frac{\partial U}{\partial t} + \frac{U}{gA}\frac{\partial A}{\partial t} \tag{4}$$

and

$$\frac{U}{gA}\frac{\partial Q}{\partial x} = \frac{U}{gA}\left(q - \frac{\partial A}{\partial t}\right) = \frac{Uq}{gA} - \frac{U}{gA}\frac{\partial A}{\partial t} \tag{5}$$

where we used the continuity equation, eq. (2) in the paper, in the middle step. Adding these two terms, the term proportional to $\partial_t A$ cancels out, and the resulting equations is given by equation (4) in the paper.

**Text S2.**

We define $\overline{p(x,t)}$, the reach-average of a hydraulic variable $p(x)$, as the result of the convolving $p(x)$ with smoothing kernel, $f(x)$:

$$\overline{p}(x,t) = \int_{-\infty}^{\infty} dx'\, f(x-x')p(x',t) = \int_{-\infty}^{\infty} dx'\, f(x')p(x-x',t) \tag{6}$$

To conserve constant values and have an associated reach scale, $L_R$, we assume averaging kernel, $f(x)$, is a function satisfying

$$\int_{-\infty}^{\infty} dx\, f(x) \;=\; 1 \tag{7}$$

$$\int_{-\infty}^{\infty} dx\, xf(x) \;=\; \frac{L_R}{2} \tag{8}$$

(the factor of 1/2 in the last equation is chosen so that $L_R$ is the reach length for uniform weighting). The hydraulic variable $p$ can then be decomposed into reach-averaged

and fluctuating components, $p(x,t) = \overline{p}(x,t) + \delta p(x,t)$, where $\delta p(x,t)$ captures all of the variability occurring at scales smaller than $L_R$. By construction, one will have that $\overline{\delta p}(x,t) = 0$; i.e., the small scale variations are zero-mean over the reach length. From the properties of the convolution, reach-averaging and differentiation commute

$$\frac{\partial \overline{p}(x,t)}{\partial t} = \overline{\frac{\partial p(x,t)}{\partial t}} \tag{9}$$

$$\frac{\partial \overline{p}(x,t)}{\partial x} = \overline{\frac{\partial p(x,t)}{\partial x}} \tag{10}$$

Therefore, if $p$ is differentiable, $\overline{p}$ is also differentiable, and the reach-averaged Saint-Venant equations are well defined.

In prior studies, (Garambois & Monnier, 2015) and (Durand et al., 2014; Yoon et al., 2016; Durand et al., 2016) assumed uniform weighting for a reach defined between $x_u$ and $x_d$, the upstream and downstream coordinates, respectively, and the weighting function, $f_U$, was given by

$$f_U(x) = \begin{cases} \frac{1}{x_d - x_u} & x_u \le x \le x_d \\ 0 & \text{Otherwise} \end{cases} \tag{11}$$

In this case, the reach-averaged steady gradually varying flow term can be integrated explicitly

$$\overline{\frac{d}{dx}\left(h(x) + \beta\frac{U^2(x)}{2g}\right)} = \frac{1}{x_d - x_u}\int_{x_u}^{x_d} dx\,\frac{d}{dx}\left(h(x) + \beta\frac{U^2(x)}{2g}\right) \tag{12}$$

$$= \frac{1}{x_d - x_u}\left(h(x) + \beta\frac{U^2(x)}{2g}\right)\Big|_{x_u}^{x_d} \tag{13}$$

Although uniform weighting agrees with the conventional meaning of reach averaging and handles control points well, smoothing kernels with better spectral properties may be preferable for modeling purposes, since the spectral leakage of the uniform window

is high. The discussion in the paper applies to general smoothing kernels, including the uniform kernel.

**Text S3.**

In this section, we provide the detailed derivation of the steps from equation (15) to equation (24) in the paper.

As in the paper, we start with

$$Q(x, \mathbf{p}) = \prod_{i=1}^{N_p} p_i^{\alpha_i}(x) \tag{14}$$

where $p_i$ is the *ith* river hydraulic parameter, and $\alpha_i$ is the corresponding exponent, and we take the friction parameter, $\rho$, to be the $i = N_p$ parameter.

Taking the logarithm of both sides and using $\log(\prod_i z_i) = \sum_i \ln z_i$ results in

$$\ln Q = \sum_{i=1}^{N_p} \alpha_i \ln(p_i) \tag{15}$$

where we have used $\ln(p_i^{\alpha_i}) = \alpha_i \ln(p_i)$. Reach averaging both sides and using the results in S2, yields

$$\overline{\ln(Q)} = \sum_{i=1}^{N_p} \alpha_i \overline{\ln(p_i)} \tag{16}$$

Because of the nonlinearity of the logarithm, $\overline{\ln p_i} \neq \ln \overline{p_i}$ in general, unless $p_i$ is constant. In fact, using Jensen's inequality (Jensen, 1906), one has that $\overline{\ln p_i} \leq \ln \overline{p_i}$. We characterize this difference through a positive indefinite variability index $\kappa_i$ defined by

$$\overline{\ln p_i} = \ln \overline{p_i} - \ln(1 + \kappa_i) \tag{17}$$

The left-hand side of equation (16)can then be expanded as

$$\overline{\ln(Q)} = \ln \overline{Q} - \ln(1 + \kappa_Q) \tag{18}$$

where $\kappa_Q$, the discharge variability index, is defined analogously to $\kappa_i$. The right-hand side of equation (16) becomes

$$\sum_{i=1}^{N_p} \alpha_i \overline{\ln(p_i)} = \sum_{i=1}^{N_p} \alpha_i \ln \overline{p_i} - \sum_{i=1}^{N_p} \alpha_i \ln(1 + \kappa_i) \tag{19}$$

Equating left and right-hand sides, moving the $\kappa_Q$ term to the right hand side, one gets

$$\ln \overline{Q} = \sum_{i=1}^{N_p - 1} \alpha_i \ln \overline{p_i} - \ln \overline{\rho} - \sum_{i=1}^{N_p} \alpha_i \ln(1 + \kappa_i) + \ln(1 + \kappa_Q) \tag{20}$$

where we have used the fact that, by convention, $\rho$ is the last parameter and $\alpha_\rho = -1$. Gathering the last three logarithms

$$\ln \overline{\rho} + \sum_{i=1}^{N_p} \alpha_i \ln(1 + \kappa_i) - \ln(1 + \kappa_Q) = \ln \left( \left[ \frac{1}{(1 + \kappa_Q)} \prod_{i=1}^{N_p} (1 + \kappa_i)^{\alpha_i} \right] \overline{\rho} \right) \tag{21}$$

Using this equation, one can write the reach-averaged discharge equation as

$$\ln \overline{Q} = \sum_{i=1}^{N_p - 1} \alpha_i \ln \overline{p_i} - \ln \tilde{\rho} \tag{22}$$

$$\tilde{\rho} = \left[ \frac{1}{(1 + \kappa_Q)} \prod_{i=1}^{N_p} (1 + \kappa_i)^{\alpha_i} \right] \overline{\rho} \equiv (1 + \kappa_T) \, \overline{\rho} \tag{23}$$

and we define the total variability index as $\kappa_T$. Taking the exponential of equation (16), we obtain

$$\overline{Q} = \frac{1}{\tilde{\rho}} \prod_{i=1}^{N_p - 1} \overline{p_i}^{\alpha_i} \tag{24}$$

which is equation (24) in the paper, as was desired.

**Text S4.**

Starting with the hyperplane equation for the parameters derived in Section (4.3)

$$\sum_{i=1}^{N_p} \alpha_i \eta_i(x) = 0 \tag{25}$$

one can obtain a set of $N_p$ equations for the variabilities and co-variabilities of the log hydraulic parameters by multiplying by $\eta_j$ and reach-averaging

$$\sum_{i=1}^{N_p} \alpha_i \overline{\eta_i \eta_j} = 0 \text{ for} 1 \leq j \leq N_p \tag{26}$$

There will be $N_p$variances, and $N_p(N_p - 1)/2$ covariabilities, so it is possible to use equation (26) to express the co-variabilities in terms of the variances for $1 < N_p \leq 3$. The case of $N_\alpha = 1$ is not consistent with the assumption of constant discharge. For $N_\alpha > 3$, the variances do not uniquely determine the co-variabilities, and there will be multiple solutions that conserve the discharge.

Since it is usually the variance of the parameters (rather than the log parameters) that is known, we use the weak fluctuation limit for equation (26) as an approximation

$$\sum_{i=1}^{N_p} \alpha_i \overline{\epsilon_i \epsilon_j} = 0 \text{ for} 1 \leq j \leq N_p \tag{27}$$

In the case when $N_p = 2$, the solution is

$$\overline{\epsilon_1 \epsilon_2} = \frac{-\left(\alpha_1 \overline{\epsilon_1^2} + \alpha_2 \overline{\epsilon_2^2}\right)}{\alpha_1 + \alpha_2} \tag{28}$$

The case of $N_p = 3$, leads to the set of equations

$$\begin{bmatrix} \alpha_2 & \alpha_3 & 0 \\ \alpha_1 & 0 & \alpha_3 \\ 0 & \alpha_1 & \alpha_2 \end{bmatrix} \begin{bmatrix} \overline{\epsilon_1 \epsilon_2} \\ \overline{\epsilon_1 \epsilon_3} \\ \overline{\epsilon_2 \epsilon_3} \end{bmatrix} = - \begin{bmatrix} \alpha_1 \overline{\epsilon_1^2} \\ \alpha_2 \overline{\epsilon_2^2} \\ \alpha_3 \overline{\epsilon_3^2} \end{bmatrix} \tag{29}$$

which can be easily inverted.

**Text S5.**

We model a constant-width periodic riffle and pool bathymetry, $Z_0(x)$, using equation (30) in the text, which has bed slope, $\partial_x Z_0$, given by equation (31). The bathymetry, illustrated in Figures (7) and (8) in the text, represents a riffle and pool sequence, of pe-

riodicity $L$, with long pools, with slope $S_p$, alternating with short riffle sequences whose slope is approximately $4S_0$. The reach averaged bathymetry is a tilted plane of slope $S_0$.

$$Z_0(x) = -S_0 x - (S_0 - S_p)\left(\frac{L}{2\pi}\right)\left[\frac{5}{3}\sin\theta + \frac{1}{2}\sin 2\theta + \frac{1}{9}\sin 3\theta\right] \tag{30}$$

$$\frac{\partial Z_0}{\partial x} = -S_0 - (S_0 - S_p)\left[\frac{5}{3}\cos\theta + \cos 2\theta + \frac{1}{3}\cos 3\theta\right] \tag{31}$$

$$\theta = \frac{2\pi}{L}x \tag{32}$$

## References

Boussinesq, J. (1877). *Essai sur la théorie des eaux courantes.* Impr. nationale.

Chaudhry, M. H. (2008). *Open-Channel Flow.* doi: 10.1007/978-0-387-68648-6

Chow, V. T. (1959). *Open channel hydraulics.* New York, McGraw-Hill.

Dingman, S. L. (2009). *Fluvial hydraulics.* Oxford University Press.

Durand, M., Gleason, C., Garambois, P.-A., Bjerklie, D., Smith, L., Roux, H., ... others (2016). An intercomparison of remote sensing river discharge estimation algorithms from measurements of river height, width, and slope. *Water Resources Research*, *52*(6), 4527–4549.

Durand, M., Neal, J., Rodriguez, E., Andreadis, K. M., Smith, L. C., & Yoon, Y. (2014). Estimating reach-averaged discharge for the River Severn from measurements of river water surface elevation and slope. *Journal of Hydrology*, *511*, 92104. doi: 10.1016/j.jhydrol.2013.12.050

Garambois, P.-A., & Monnier, J. (2015). Inference of effective river properties from remotely sensed observations of water surface. *Advances in Water Resources*, *79*, 103–120. doi: 10.1016/j.advwatres.2015.02.007

Jensen, J. L. W. V. (1906). Sur les fonctions convexes et les inégalités entre les valeurs moyennes. *Acta Math.*, *30*, 175–193. Retrieved from https://doi.org/10.1007/BF02418571 doi: 10.1007/BF02418571

Ponce, V. M., & Simons, D. B. (1977). Shallow wave propagation in open channel flow. *Journal of the Hydraulics Division*, *103*(12), 1461–1476. Retrieved from http://ponce.sdsu.edu/shallow_wave_propagation_in_open_channel_flow.html

Yoon, Y., Garambois, P.-A., Paiva, R., Durand, M., Roux, H., & Beighley, E. (2016). Improved error estimates of a discharge algorithm for remotely sensed river measurements: Test cases on Sacramento and Garonne Rivers. *Water Resources Research*, *52*. doi: 10.1002/2015WR017319
